# Single‐Nucleus Transcriptomic Atlas of Human Vellus Hair Pilosebaceous Units Reveals Age‐Associated Remodeling

**DOI:** 10.1002/advs.78048

**Published:** 2026-09-29

**Authors:** Ya'nan Li, Zhuoqiong Qiu, Xiaoyu Pan, Geyang Xu, Shanshan Peng, Ronghui Zhu, Qiuyang Luo, Xiaokai Fang, Xu Yao, Wei Li

**Affiliations:** ^1^ Department of Dermatology, Shanghai Institute of Dermatology, National Clinical Research Center for Aging and Medicine, Huashan Hospital Fudan University Shanghai People's Republic of China; ^2^ Department of Dermatology, Second Affiliated Hospital Zhejiang University School of Medicine Hangzhou People's Republic of China; ^3^ Department of Computer Science University of North Carolina At Chapel Hill Chapel Hill USA; ^4^ Department of Dermatology, Wuhan No. 1 Hospital, Tongji Medical College Huazhong University of Science and Technology Wuhan People's Republic of China; ^5^ Hubei Province & Key Laboratory of Skin Infection and Immunity Wuhan People's Republic of China; ^6^ Department of Allergy and Rheumatology, Jiangsu Key Laboratory of Molecular Biology For Skin Diseases and STIs, Hospital For Skin Diseases, Institute of Dermatology Chinese Academy of Medical Sciences and Peking Union Medical College Nanjing People's Republic of China

**Keywords:** hair follicle, melanocyte, sebaceous gland, skin aging, transcriptome

## Abstract

The pilosebaceous units (PSUs) are essential micro‐organs for skin homeostasis, yet human vellus hair‐associated PSUs remain largely understudied. Here, we present a single‐nucleus transcriptomic atlas of PSUs from the back skin of healthy young and aged males, revealing extensive age‐associated cellular and transcriptional remodeling within this niche. We show that aging is accompanied by reduced relative representation of bulge hair follicle stem cells, together with altered regeneration‐associated transcriptional programs. Sebocytes display enhanced androgen responsiveness coupled with increased lipid biosynthetic activity during aging. Concurrently, an ion channel‐enriched follicular population shows increased representation with age and exhibits stress‐ and inflammation‐associated transcriptional features. In parallel, an innate immune‐like melanocyte subset shows reduced relative representation during aging, coinciding with changes in inferred melanocyte–immune communication. Notably, immune subsets in aged PSUs display coordinated changes in metabolic pathway signatures, suggesting an association between immune remodeling and metabolic state. Together, our findings highlight the aged PSU as a focal site where epithelial, immune, and metabolic alterations converge during skin aging. This work establishes a comprehensive single‐nucleus atlas of human vellus PSU aging and provides a framework for understanding how aging reshapes pilosebaceous niche biology, with potential implications for age‐associated cutaneous inflammation and dysfunction.

## Graphical abstract

Human vellus pilosebaceous units (PSUs) remain uncharted. This single‐nucleus atlas reveals coordinated remodeling of the aging PSU niche: reduced bulge stem cell representation with altered regenerative programs, increased representation of a stress‐responsive channel^+^ epithelial state, enhanced androgen‐responsive sebaceous programs, reduced representation of an immune‐associated melanocyte state, and immune compositional and signaling remodeling, with implications for age‐associated cutaneous inflammation and dysfunction.

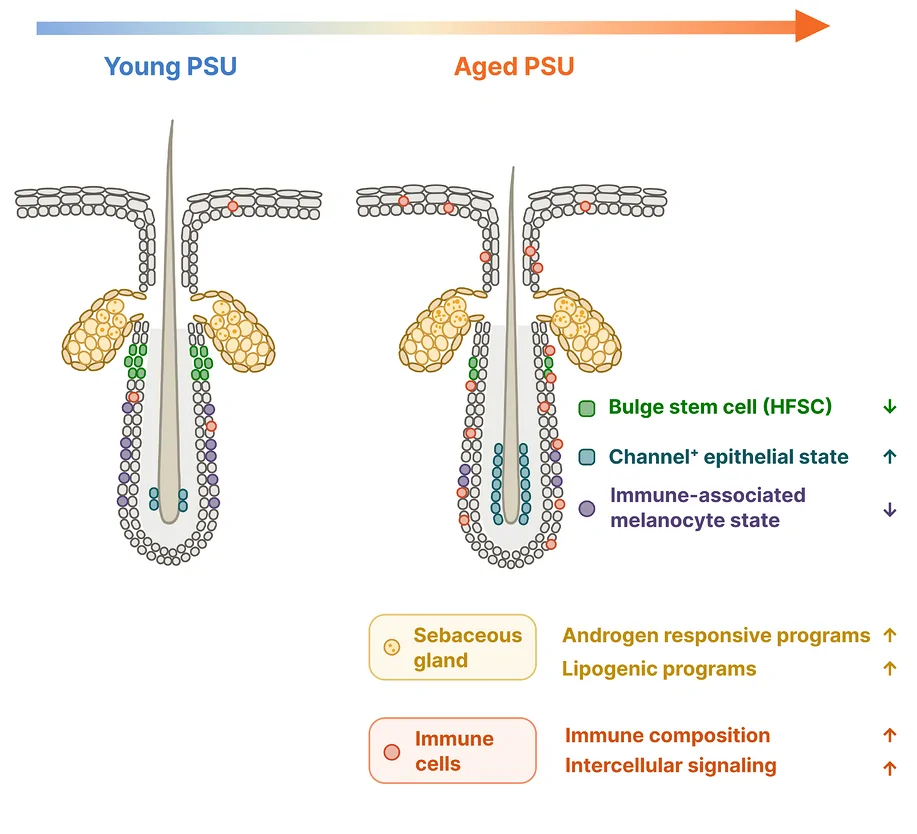

## 1 Introduction

The pilosebaceous unit (PSU) is a complex skin mini‐organ composed primarily of the hair follicle and sebaceous gland, expanding the epithelial surface area of the skin from 2 m^2^ to approximately 25 m^2^ [1]. Beyond hair production, PSUs actively contribute to cutaneous homeostasis through lipid secretion, antimicrobial defense [2], regulation of skin microbiota [3, 4], and maintenance of epithelial stem cell reservoirs [5]. Previous studies of human hair biology have focused predominantly on terminal hair follicles of the scalp because of their relevance to hair cycle dynamics [6], stem cell biology [7, 8], and conditions like alopecia [9] and hair graying [10]. In contrast, vellus hair follicles have received comparatively little attention despite constituting the predominant follicular appendage across most human skin surfaces. Unlike terminal follicles, which primarily contribute to visible hair growth, vellus hair‐associated PSUs form a ubiquitous epithelial–sebaceous–immune network crucial for barrier maintenance, tissue homeostasis, and local immune regulation. Deciphering the cellular organization of vellus PSUs is therefore essential for understanding skin physiology beyond hair biology alone.

PSUs are also deeply implicated in skin aging [11, 12]. Hair follicles harbor stem cell populations that coordinate tissue regeneration and interact extensively with surrounding epithelial, immune, and mesenchymal compartments. Previous studies have shown that age‐associated deterioration of the stem cell niche compromises hair follicle stem cell (HFSC) function and regenerative capacity [13]. In parallel, sebaceous gland activity, microbial colonization, and immune surveillance undergo substantial remodeling during aging, collectively influencing the follicular microenvironment [14, 15, 16, 17]. Because vellus PSUs are distributed across nearly the entire body surface, age‐associated alterations within this niche may have implications for clinically relevant aging phenotypes, including barrier dysfunction, xerosis, impaired tissue repair, and chronic low‐grade inflammation. Understanding how aging reshapes this niche is therefore important for elucidating the cellular basis of skin aging.

Single‐cell technologies have substantially advanced our understanding of epidermal and hair follicle biology in both mice and humans [18, 19, 20, 21, 22, 23]. Recent studies have generated transcriptomic atlases of human scalp hair follicles and investigated processes such as stem cell maintenance [21, 24], and hair graying [10]. However, most studies have focused on terminal hair follicles, whereas the cellular architecture and age‐associated remodeling of human vellus PSUs remain poorly defined. Given the cellular heterogeneity of the PSU and the complexity of age‐associated tissue remodeling, a high‐resolution characterization of human vellus PSUs is still lacking.

Here, we generated a single‐nucleus RNA‐sequencing (snRNA‐seq) atlas of human vellus hair PSUs from the back skin of young and aged male individuals, using nuclear profiling to minimize dissociation‐associated cell loss inherent to single‐cell RNA sequencing (scRNA‐seq). Comparative analyses revealed marked age‐associated changes across multiple PSU compartments, including sebaceous glands, melanocytes, and immune cells. We observed age‐associated alterations in hair follicle stem cell populations and immune niches, alongside increased relative representation of a channel^+^ epithelial state in aged PSUs. This state exhibited stress‐responsive transcriptional programs and altered inferred intercellular communication. In addition, we identified an innate immune‐like melanocyte subset with reduced relative representation in aged PSUs. Together, these findings provide a comprehensive cellular framework for understanding human vellus PSU aging and reveal previously unappreciated features of niche remodeling during skin aging.

## 2 Results

### 2.1 Single‐Cell Landscape of the Young and Aged Pilosebaceous Unit

To map the cellular architecture of human vellus hair PSUs and assess age‐associated changes, we performed transcriptomic profiling of back skin biopsies from 3 young (20–40 years old, scRNA‐seq and snRNA‐seq) and 6 aged (60–93 years old, snRNA‐seq) male donors (Figure 1a; Figure S1a). Our analyses focused on shared age‐associated transcriptional remodeling within defined cell populations, rather than on inter‐individual or sex‐specific variability. Principal component analysis (PCA) of pseudobulk data revealed age as the dominant source of variation (PC1), clearly separating young and aged PSUs by transcriptional profiles (Figure 1b). Comparison with Genotype‐Tissue Expression (GTEx) age‐associated signatures revealed concordant enrichment of aged skin programs and attenuation of young skin signatures in the aged PSU (Figure 1c).

**FIGURE 1:**
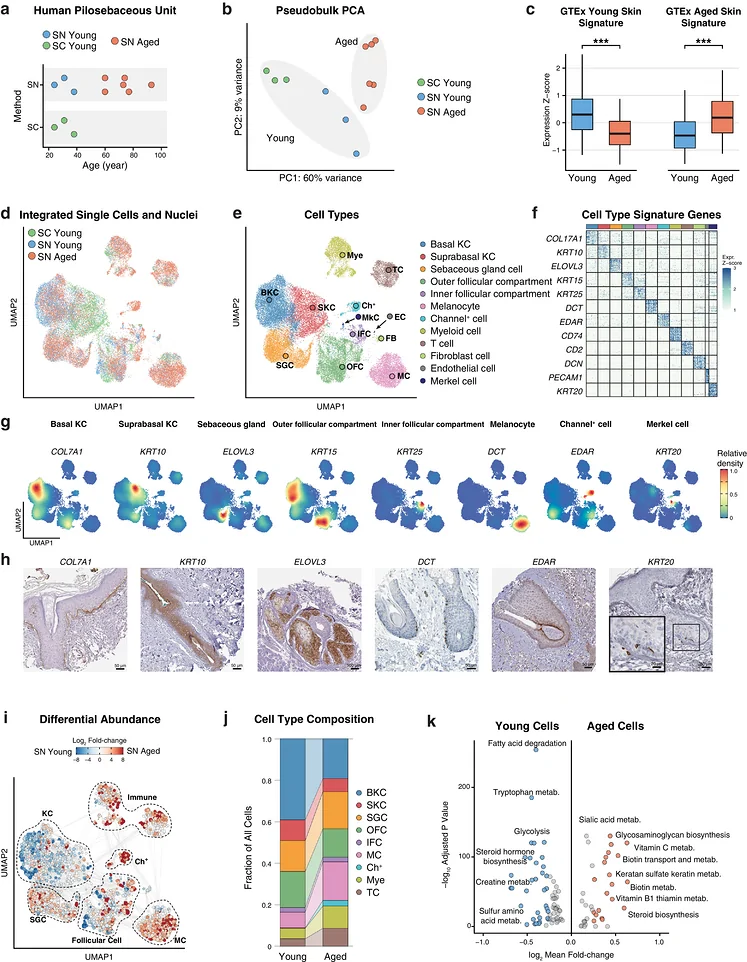
Age‐associated cellular reprogramming in human pilosebaceous units revealed by single‐nucleus transcriptomics. (a) Overview of donor age distribution of the cohort and profiling strategies. SN, single‐nucleus; SC, single‐cell. (b) Principal component analysis (PCA) of pseudobulk analysis of all samples. (c) Box plot showing per‐cell expression z‐scores of skin aging signature genes derived from the Genotype‐Tissue Expression (GTEx) database. ^***^: *p* < 0.001, by two‐tailed Student's *t*‐test. Box plots indicate median (center line), 25th–75th percentiles (bounds of box), and whiskers extend to 1.5× IQR. (d) Uniform manifold approximation and projection (UMAP) representation of integrated snRNA‐seq and scRNA‐seq data, colored by donor age group and data type. (e) UMAP colored by major PSU cell clusters. (f) Heatmap of cell type‐enriched genes with representative signature genes indicated on the left; each column represents a cell type shown in (e). (g) Nebulosa density plots showing representative signature gene expression patterns across the dataset. (h) Representative immunohistochemistry images from the Human Protein Atlas (HPA) [26] illustrating the anatomical localization of major PSU cell layers. Images are reproduced under the Creative Commons Attribution 4.0 International License (CC BY 4.0). Image credit: Human Protein Atlas. Image‐specific source information is provided in Table S1. Scale bars as indicated. (i) Milo differential abundance testing across all single nuclei. Nodes represent neighborhoods colored by log_2_ fold‐change between age groups; edges indicate shared cell numbers between neighborhoods. (j) Composition of major cell types identified in the snRNA‐seq data comparing two age groups. (k) Pathway‐level comparison of metabolic programs highlighting global metabolic shifts between young and aged PSUs.

Integrated analysis of scRNA‐seq and snRNA‐seq datasets, corrected for batch effects, showed comparable distributions across individual libraries, and revealed both age‐related heterogeneity and technical differences between profiling methods (Figure 1d). Notably, consistent quality control metrics across all libraries supported the interpretation that the observed cell states were unlikely to arise solely from technical variation (Figure S1b). Twelve PSU cell types were identified using reference‐based annotation and established markers (Figure 1e), including basal keratinocyte (BKC, *COL17A1* ^+^), suprabasal keratinocyte (SKC, *KRT10* ^+^), sebaceous gland cell (SGC, *ELOVL3* ^+^), outer follicular compartment (OFC, *KRT15* ^+^), inner follicular compartment (IFC, *KRT25* ^+^), melanocyte (MC, *DCT* ^+^), myeloid cell (Mye, *CD74* ^+^), T cell (TC, *CD2* ^+^), fibroblast cell (FB, *DCN* ^+^), endothelial cell (EC, *PECAM1* ^+^), Merkel cell (MkC, *KRT20* ^+^) and channel^+^ cell (Ch^+^, *ATP1B3* ^+^ *EDAR* ^+^) (Figure 1e–h; Figure S1c). Among these, we observed an ion channel‐enriched population, here referred to as “channel^+^ cells”, which exhibited a transcriptional profile distinct from canonical epidermal or follicular cell types and aligned with the previously unannotated “unknown” cluster in a prior whole‐skin reference atlas [25]. Integration of scRNA‐seq and snRNA‐seq data verified consistent identification of major PSU populations (Figure S1d,e), while Milo differential abundance analysis indicated differences between profiling modalities, suggesting that the observed age‐associated differences were unlikely to be solely explained by profiling modality (Figure S1f). Representative signature gene expression patterns and corresponding protein expression data from the Human Protein Atlas (HPA) [26] supported the annotation and anatomical localization of major PSU populations (Figure 1g,h). Metabolic pathway analysis revealed cell‐type‐specific enrichments, such as lipid metabolism in SGCs and nicotinamide metabolism in channel^+^ cells (Figure S1g,h).

We next focused on conserved differences between young and aged PSUs. To mitigate technical variance, all comparative analyses were performed using the snRNA‐seq dataset. Milo differential abundance testing and composition analysis revealed significant and reproducible shifts in cell populations between young and aged PSUs (Figure 1i,j; Figure S1d), including increased relative representation of SGCs, melanocytes, and immune compartments in aged PSUs. We also observed age‐associated changes in inferred metabolic activities, including reduced activity of energy metabolism‐related pathways (e.g., fatty acid degradation and glycolysis) and enhanced activity of biotin and vitamin C metabolism pathways in cells from aged PSUs (Figure 1k; Figure S1i). These findings provide a high‐resolution cellular and metabolic reference landscape of the human PSU during aging, highlighting coordinated age‐associated remodeling across epithelial, immune, and metabolic compartments.

### 2.2 Age‐Associated Remodeling of Follicular Epithelial States and Regenerative Programs

To investigate how aging remodels follicular epithelial compartments within the PSU, we focused on the outer and inner follicular compartments (OFC and IFC) and performed subclustering analysis to resolve their cellular heterogeneity. The OFC was resolved into six transcriptionally distinct states (OFC1‐OFC6) (Figure 2a,b). Representative marker expression patterns, together with HPA‐based immunohistochemistry (IHC) and our own immunostaining, supported the spatial annotation of these OFC states (Figure 2c,d). OFC1, expressing *PTHLH*, corresponded to outer root sheath (ORS) suprabasal cells (ORS‐SB) while retaining certain HFSC features [18]. OFC2 was enriched for canonical HFSC markers (*CXCL14*, *KRT15*, *FOXC1*, and *COL17A1*), identifying it as a quiescent bulge HFSC population [27]. OFC3 expressed *LGR6* and corresponded to an upper ORS transitional state associated with the isthmus region [28]. OFC4 expressed HFSC regulators (*LGR4*, *LGR5*, *TAGLN*, and *SLC1A3*), consistent with an activated progenitor state in the lower ORS [19, 29]. OFC5, a *KRT6B* ^+^ cell state co‐expressing HFSC transcription factors (*SOX9* and *HES1*), corresponded to a lower ORS anchoring population [30]. OFC6 exhibited elevated Wnt/Shh pathway activity together with lower ORS markers, defining a Wnt/Shh‐high transitional state (Figure S2a). We next evaluated the age‐related dynamics within the OFC. Aging was associated with reduced proportions of OFC2 and OFC5, accompanied by the increased representation of OFC3 and OFC6 populations (Figure 2e). To further characterize progenitor states within the OFC, we quantified quiescent HFSC and primed progenitor signatures using established follicular stem cell gene programs [27]. Quiescent scores were highest in OFC2, whereas primed progenitor signatures were enriched in OFC4 and OFC6 (Figure 2f), supporting the annotation of OFC2 as a quiescent stem cell state and OFC4/OFC6 as downstream progenitor‐associated states. Based on its enrichment for quiescent HFSC signatures, OFC2 was used as the root state for Slingshot trajectory inference, which reconstructed three major transcriptomic branches associated with OFC1, OFC5, and OFC6, respectively (Figure 2g). Progressive changes in quiescent HFSC marker expression and acquisition of branch‐associated markers further supported the inferred transcriptional relationships along each trajectory (Figure S2b). Furthermore, aged OFCs exhibited reduced quiescent HFSC signatures and increased primed progenitor signatures (Figure S2c), accompanied by reduced representation of early OFC states and a redistribution toward later pseudotime‐associated states (Figure S2d).

**FIGURE 2:**
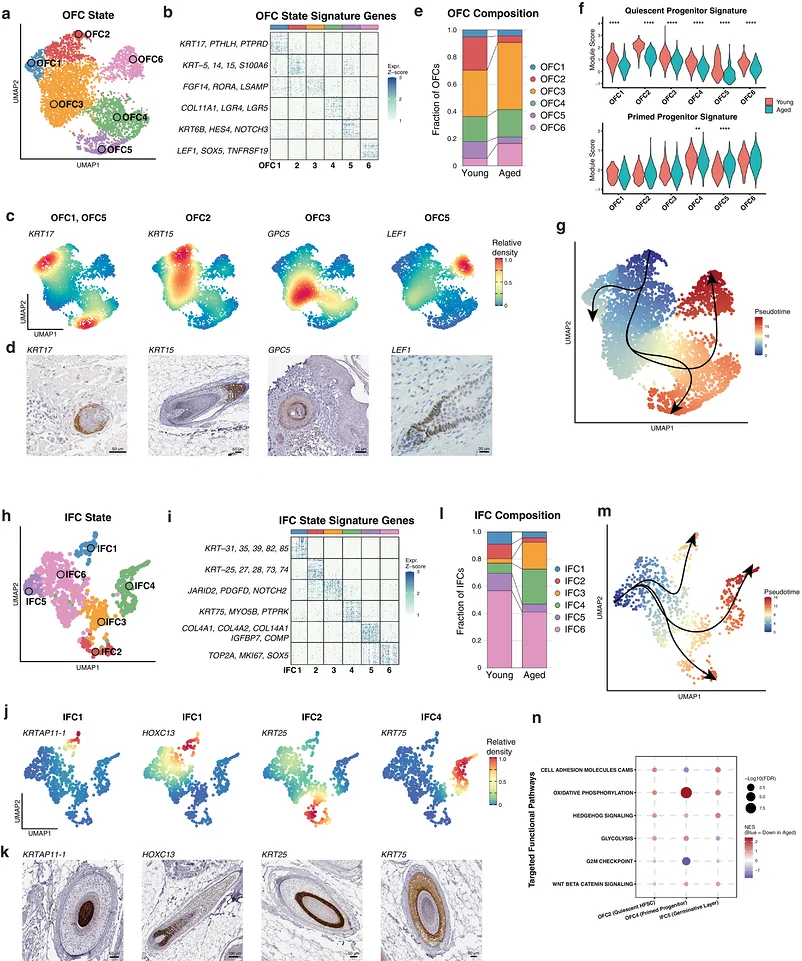
Remodeling of follicular epithelial states and regenerative programs during aging. (a) UMAP plot of the Outer Follicular Compartment (OFC) subset resolving six transcriptional states (OFC1‐OFC6). (b) Heatmap of OFC state signature genes (scaled Z‐scores) with representative markers listed on the left. (c) Nebulosa density plots showing representative gene expression patterns within OFCs. (d) Representative immunohistochemistry images of human PSUs from the HPA [26] (validating OFC1‐3) and our staining (validating OFC5). Images are reproduced under the Creative Commons Attribution 4.0 International License (CC BY 4.0). Image credit: Human Protein Atlas. Image‐specific source information is provided in Table S1. Scale bars as indicated. (e) Proportional abundance of OFC states in young versus aged groups. (f) Quiescent hair follicle stem cell (HFSC) and primed progenitor signature scores across OFC states, calculated using established follicular stem cell gene programs. (g) Slingshot pseudotime trajectory inferred for the OFC population using OFC2 (quiescent bulge HFSCs) as the root; cells colored by pseudotime rank. (h) UMAP plot of the Inner Follicular Compartment (IFC) subset resolving six transcriptional states (IFC1‐IFC6). (i) Heatmap of IFC state signature genes (scaled Z‐scores) with representative marker genes listed on the left. (j) Nebulosa density plots showing representative signature gene expression patterns within IFCs. (k) Representative immunohistochemistry images of human PSUs from the HPA [26] (validating IFC1, IFC2, and IFC4 markers). Images are reproduced under the Creative Commons Attribution 4.0 International License (CC BY 4.0). Image credit: Human Protein Atlas. Image‐specific source information is provided in Table S1. Scale bars as indicated. (l) Proportional abundance of IFC states in young versus aged groups. (m) Slingshot pseudotime trajectory inferred for the IFC population using IFC5 (germinative layer cells) as the root; cells colored by pseudotime rank. (n) Gene set enrichment analysis comparing young and aged progenitor‐associated compartments (OFC2, OFC4, and IFC5).

The IFC was similarly resolved into 6 transcriptional states (IFC1‐IFC6) (Figure 2h,i). Marker gene expression and HPA‐based IHC validation identified IFC1 as hair shaft (HS) cells, IFC2 and IFC3 as inner root sheath (IRS) states, IFC4 as a companion layer‐like differentiated state characterized by *KRT6* expression [31] (Figure 2j,k). IFC5 was characterized by high expression of *LGR5* and *CDH3* and was enriched for extracellular matrix organization, basement membrane organization, and focal adhesion pathways, consistent with a germinative layer (GL) identity [32, 33]. *MKI67* ^+^ *TOP2A* ^+^ IFC6 was enriched for cell cycle and DNA replication, consistent with proliferative matrix transit‐amplifying cells (TACs) [19]. Compositional analysis revealed marked age‐associated remodeling within the IFC compartment. IFC5 and IFC6 represented the predominant populations in young PSUs but were substantially depleted in aged PSUs, whereas IFC3 and IFC4 expanded with aging (Figure 2l). Trajectory inference using IFC5 as the root reconstructed three major transcriptional trajectories corresponding to transcriptional continua toward HS‐, IRS‐, and companion layer‐associated states, respectively (Figure 2m). Lineage‐specific markers changed progressively along each branch, supporting the organization of IFC states along distinct transcriptional continua (Figure S2e). Consistent with these observations, global pseudotime density analysis revealed a marked redistribution of aged IFCs toward later lineage‐associated states, whereas young IFCs were predominantly enriched in early progenitor‐associated states (Figure S2f).

To determine whether these compositional changes were accompanied by transcriptional pathway alterations, we performed pathway enrichment analysis in progenitor‐associated compartments. Aged OFC2 cells exhibited reduced WNT/β‐catenin signaling signatures together with increased oxidative phosphorylation and glycolytic programs (Figure 2n). In parallel, aged OFC4 cells displayed marked suppression of G2M checkpoint activity accompanied by a strong increase in oxidative phosphorylation, suggesting alterations in proliferative and metabolic programs. Similarly, IFC5 germinative layer cells showed downregulation of cell cycle‐associated programs, including G2M checkpoint signaling (Figure 2n). Together, these findings suggest that age‐associated remodeling of follicular progenitor compartments is accompanied by altered regenerative signaling signatures, attenuation of cell‐cycle‐associated programs, and shifts in metabolic pathway activities.

Finally, analysis of the interfollicular epidermis (IFE) keratinocyte population identified two basal keratinocyte subsets (BKC1/2, *KRT14* ^+^ *KRT15* ^+^), a proliferative subset (BKC4, *TOP2A* ^+^ *MKI67* ^+^), and two suprabasal populations distinguished by *SPINK5* and *DSG1* (granular vs spinous SKCs; Figure S3a,b). Total keratinocyte (KC) abundance was significantly reduced in aged PSUs, whereas the proportional distribution of individual subsets remained largely stable (Figure S3c), consistent with previous observations [34]. Nonetheless, aged KCs exhibited functional transcriptomic alterations, characterized by reduced enrichment of fatty acid degradation pathways and decreased expression of *POSTN* and barrier‐related genes such as *KRT15*, *DSG1*, and *PERP* (Figure S3d–g), suggesting age‐associated attenuation of epidermal barrier‐related programs.

### 2.3 Cellular Heterogeneity and Age‐Associated Functional Adaptation in Sebaceous Glands

As a key component of the PSU, the sebaceous gland (SG) is attached to the hair follicle (HF) and opens into the upper follicle, coordinating their development and activities. We identified four transcriptionally distinct SGC subsets (Figure 3a). SGC1, marked by *TOP2A* and *MKI67*, represented actively proliferating cells. SGC2, a *KRT15* ^+^ population located at the apical part of the SG, likely corresponded to SG precursors. SGC3 and SGC4, expressing *PPARG* and *CD36*, respectively, were associated with mature SG function (Figure 3b). Metabolic pathway scoring revealed enrichment of lipid, triglyceride, and fatty acid metabolism in SGC3 and SGC4 cells, consistent with their mature sebocyte transcriptional profiles (Figure S4a). In contrast, SGC1 and SGC2 showed transcriptional similarity to keratinocytes, indicating early or precursor states (Figure S4b). To investigate transcriptional relationships among SGC states, we performed trajectory inference using the progenitor‐like SGC2 population as the root. The resulting trajectory reconstructed a major transcriptional continuum extending from SGC2 toward mature sebocyte states (SGC3–SGC4), together with a separate proliferative branch associated with SGC1 (Figure 3c).

**FIGURE 3:**
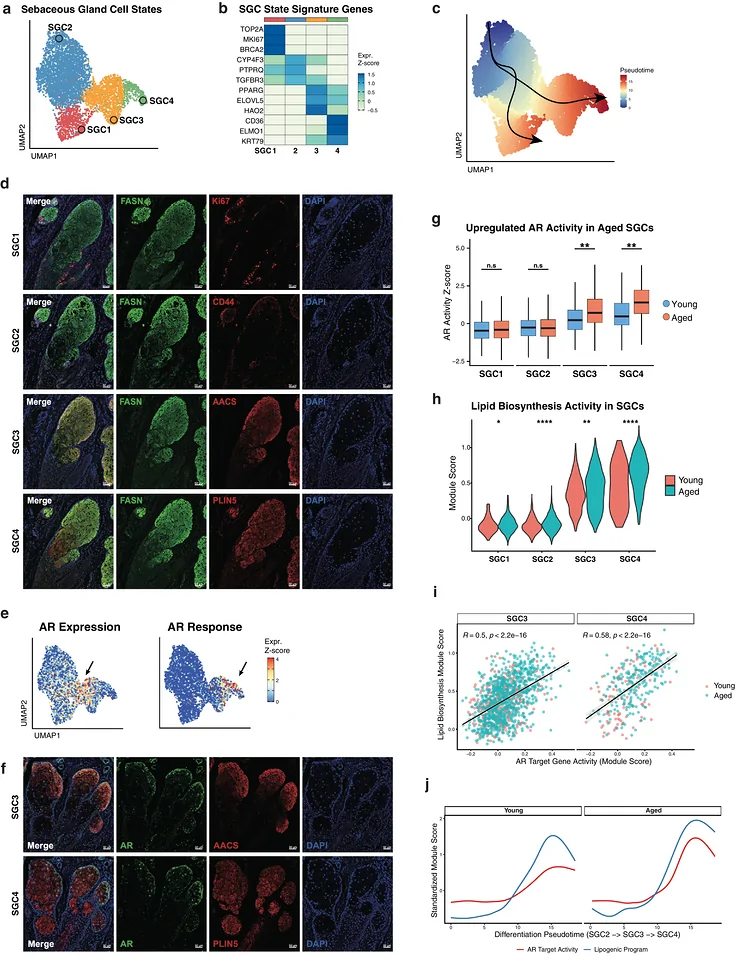
Spatiotemporal mapping of sebocyte differentiation and coordinated androgen‐lipogenic programs during aging. (a) UMAP plot of sebaceous gland cells (SGCs) resolving four transcriptional states (SGC1‐SGC4). (b) Heatmap of representative SGC state signature genes. (c) Slingshot pseudotime trajectory anchored on the *KRT15* ^+^ SGC2 precursor state, illustrating a proliferative branch associated with SGC1 and a differentiation trajectory leading toward mature sebocyte states (SGC3/4). (d) Immunofluorescence images showing representative SGC marker expression. The pan‐sebocyte marker FASN (green) outlines the sebaceous gland architecture, highlighting the spatial restriction of specific subsets (red). Scale bars, 50 µm. (e) UMAP feature plots showing androgen receptor (*AR*, left) and *AR* response gene module expression (right). (f) Immunofluorescence staining of AR. AR expression is restricted to the nuclei of differentiating sebocytes, validating the snRNA‐seq‐inferred SGC3 state. Scale bars, 50 µm. (g) Box plot of *AR* activity scores across SGC states stratified by age; ^**^: *p* < 0.01, by two‐tailed Wilcoxon rank‐sum test; ns: not significant. (h) Violin plots comparing lipid biosynthesis module scores across SGC states stratified by age; ^*^: *p* < 0.05, ^**^: *p* < 0.01, ^****^: *p* < 0.0001, by two‐tailed Wilcoxon rank‐sum test. (i) Scatter plots showing positive correlations (Pearson correlation) between AR target gene activity and lipid biosynthesis scores in mature sebocytes (SGC3 and SGC4). (j) Dynamic changes in AR‐responsive transcriptional activity (red) and lipid biosynthesis programs (blue) along the sebocyte differentiation trajectory, showing coordinated activation during sebocyte maturation.

To spatially map these transcriptomically defined subpopulations, we utilized a panel of stage‐specific markers selected for spatial resolution, extending beyond the representative genes displayed in Figure 3b. Immunofluorescence (IF) co‐staining was performed using the canonical pan‐sebocyte marker *FASN* to outline the gland architecture. Within this framework, Ki67^+^ SGC1 localized to the basal layer, *KRT15* ^+^ *CD44* ^+^ SGC2 to the apical periphery, and *AACS* ^+^ *FASN* ^+^ SGC3 and *PLIN5* ^+^ SGC4 were positioned progressively toward the SG duct (Figure 3d). This spatial pattern is consistent with the canonical maturation process of sebocytes, where proliferative and precursor‐like populations occupy peripheral regions and mature sebocytes localize toward the duct [35], supporting the inferred transcriptional transitions among SGC states. Notably, the relative abundance of SGC subsets did not differ significantly between young and aged PSUs, suggesting that age‐associated changes in SGCs are primarily reflected at the transcriptional level rather than in major shifts of subset composition (Figure S4c).

We next examined signaling pathways driving SGC activation, focusing on androgen, a well‐known stimulator of sebum secretion [36]. Androgen receptor (*AR*) expression was highest in SGC3, while downstream *AR* target genes were most enriched in SGC4 (Figure 3e). Immunofluorescence staining confirmed prominent nuclear AR protein localization within the differentiation zone corresponding to SGC3, with reduced signal in terminally differentiated sebocytes (SGC4) (Figure 3f). Quantification of AR‐response scores revealed significantly increased androgen‐responsive activity in aged sebocytes compared with young controls (Figure 3g; Figure S4d). Notably, lipid biosynthesis signatures were also significantly increased in aged mature sebocytes (SGC3 and SGC4) (Figure 3h).

To further evaluate the relationship between androgen‐responsive and lipogenic programs, we examined their association at the single‐cell level. AR target scores exhibited a strong positive correlation with lipid biosynthesis scores in both SGC3 and SGC4 populations (Figure 3i). Furthermore, along the sebocyte pseudotime trajectory, AR‐responsive transcriptional signatures and lipid biosynthesis programs increased in a highly coordinated manner and reached peak levels in late‐stage sebocytes (Figure 3j). Together, these findings suggest that enhanced androgen‐responsive transcriptional programs in aged sebocytes are coupled with increased lipogenic programs and may reflect an age‐associated adaptation of sebocyte lipid metabolism.

### 2.4 An Age‐Associated Channel^+^ Epithelial State with Altered Transcriptional and Signaling Networks

During reference‐assisted annotation of PSU cell populations, we identified a transcriptionally distinct cluster that could not be assigned to established epidermal or follicular lineages (Figure S1c). To determine whether this population represented a reproducible cellular state rather than a dataset‐specific artifact, we re‐analyzed a published human skin atlas dataset [25], and identified a discrete *ATP1B3* ^+^ *GJB6* ^+^ cluster that had remained unannotated in the original study (Figure 4a; Figure S5a,b). This population exhibited a highly concordant transcriptional signature with our undefined population (Figure 4b; Figure S5c), supporting the existence of a consistent cellular state across independent cohorts and analytical pipelines. Composition analysis revealed a consistent enrichment of this population in aged skin across both datasets (Figure 4c). Furthermore, re‐analysis of an independent psoriasis dataset [37] identified a highly similar population that showed increased representation in psoriatic lesions relative to healthy skin from multiple anatomical sites (Figure S5d,e). Together, these cross‐cohort validations indicate that increased representation of this cellular state is reproducibly associated with physiological aging and is also observed in inflammatory skin disease contexts.

**FIGURE 4:**
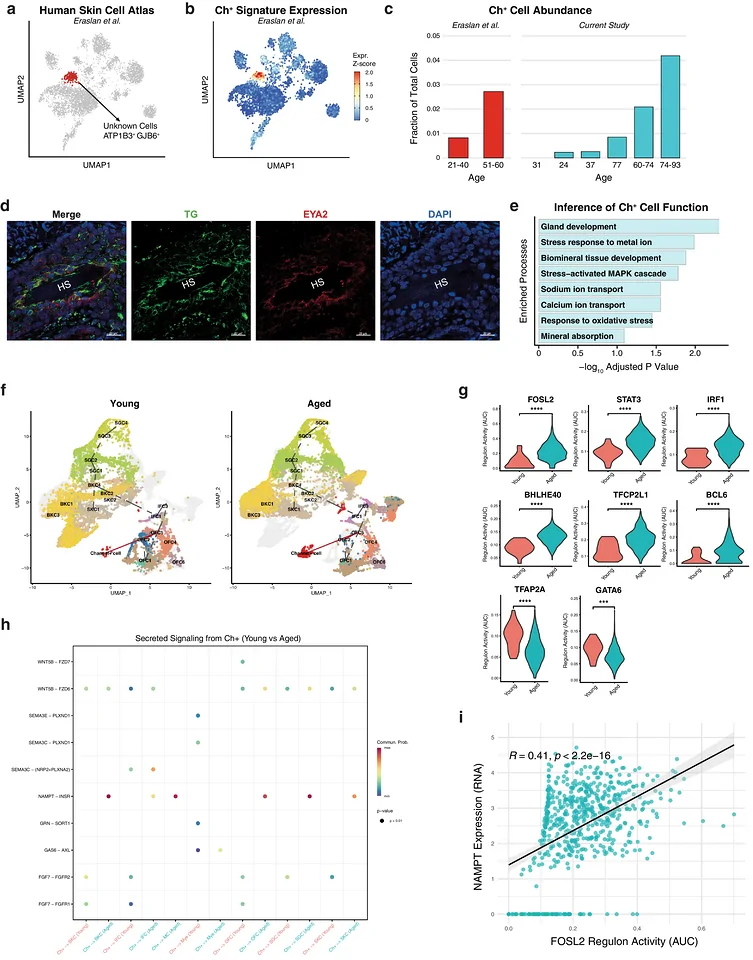
Age‐associated channel^+^ epithelial cells exhibit transcriptional remodeling and altered intercellular communication. (a) UMAP plot of the re‐analyzed Human Skin Cell Atlas (Eraslan et al.), highlighting the unannotated cluster enriched for *ATP1B3* and *GJB6* (red). (b) Feature plot showing the expression score of the Ch^+^ cell signature gene set projected onto the Human Skin Atlas dataset. (c) Bar plot showing Ch^+^ cell abundance stratified by age groups in the Human Skin Cell Atlas (red) and the current dataset (blue). (d) Immunofluorescence co‐staining of Ch^+^ marker EYA2 (red) and TG (green) with DAPI nuclear staining (blue) in a transverse section of a human hair follicle. EYA2^+^TG^+^ cells are detected in an epithelial layer immediately adjacent to the central, anucleated hair shaft (HS). Scale bars, 20 µm. (e) Significantly enriched Gene Ontology (GO) Biological Processes among Ch^+^‐enriched genes (Benjamini‐Hochberg adjusted *p* values). (f) Slingshot pseudotime trajectory analysis of epithelial lineages stratified by young and aged conditions. (g) Violin plots showing SCENIC regulon activity scores (AUC) of selected regulons (*FOSL2*, *STAT3*, *IRF1*, *BHLHE40*, *TFCP2L1*, *BCL6*, *TFAP2A*, *GATA6*) in Ch^+^ cells across young and aged groups; ^****^: *p* < 0.0001, by two‐tailed Wilcoxon rank‐sum test. (h) Bubble plot of age‐stratified CellChat analysis showing inferred outgoing secreted signaling pathways originating from Ch^+^ cells. (i) Scatter plot showing the correlation between *FOSL2* regulon activity and *NAMPT* expression across individual cells. *R* represents the Spearman correlation coefficient.

To further characterize the identity and inferred functional features of this population, we examined its spatial localization and transcriptional characteristics. Immunofluorescence staining demonstrated the presence of EYA2^+^TG^+^ cells in a narrow epithelial layer immediately adjacent to the hair shaft, consistent with localization to the innermost follicular epithelium (Figure 4d). Functional enrichment analysis of channel^+^ signature genes revealed overrepresentation of biological processes related to ion transport, metal ion homeostasis, oxidative stress adaptation, and transmembrane signaling (Figure 4e). Based on this prominent ion channel and transport‐related signature, we adopted the term “channel^+^ cells” for subsequent analyses. Together, these features suggest that channel^+^ cells represent a specialized epithelial state with transcriptional programs consistent with the unique physicochemical environment of the inner follicular compartment.

To examine the transcriptional relationship and differentiation status of channel^+^ cells, we reconstructed epithelial trajectories using Slingshot. Channel^+^ cells occupied a distinct, blind‐ended side branch within the reconstructed epithelial trajectory, branching from a trajectory region enriched for OFC2 cells, with channel^+^ cells being more represented within this branch in aged skin (Figure 4f). Consistent with this trajectory configuration, CytoTRACE analysis assigned channel^+^ cells an intermediate differentiation score between progenitor‐like and terminally differentiated epithelial populations (Figure S5f). These findings support the interpretation that channel^+^ cells represent a remodeled epithelial state rather than an independent developmental lineage. Single‐Cell Regulatory Network Inference and Clustering (SCENIC) analysis further revealed transcriptional remodeling in channel^+^ cells. Compared with other epithelial populations, channel^+^ cells showed reduced inferred activity of epithelial homeostasis‐associated regulons, including *TFAP2A* and *GATA6*, accompanied by increased inferred activity of stress‐responsive and inflammatory regulons such as *FOSL2*, *BHLHE40*, *STAT3*, and *IRF1* (Figure 4g; Figure S5g). These regulatory features are consistent with a stress‐responsive epithelial state enriched in aged PSU tissues.

Given the age‐associated enrichment and stress‐associated transcriptional profile of this channel^+^ epithelial state, we investigated its intercellular communication properties using age‐stratified CellChat analysis. Overall, communication originating from channel^+^ cells was remodeled with aging, characterized by an increased number of inferred interactions but reduced overall interaction strength (Figure S5h), together with altered incoming secreted signaling received by channel^+^ cells (Figure S5i). At the pathway level, channel^+^ cells exhibited age‐dependent alterations in inferred ligand‐receptor signaling programs. Specifically, channel^+^ cells showed reduced inferred *FGF7*‐related communication and enhanced *NAMPT*‐associated signaling, consistent with a shift toward a stress‐associated communication profile (Figure 4h; Figure S5j). Consistently, the inferred regulon activity of the stress‐responsive transcription factor *FOSL2* showed a significant positive correlation with *NAMPT* expression at the single‐cell level (Figure 4i). Together, these findings support the characterization of channel^+^ cells as an age‐associated epithelial state with transcriptional and signaling remodeling, stress‐responsive regulatory programs, and altered inferred intercellular communication within the PSU niche.

### 2.5 Age‐Associated Relative Decline of an Innate Immune‐Like Melanocyte State

Melanocytes are key for skin and hair pigmentation and protection against ultraviolet (UV)‐induced DNA damage [38]. While previous studies distinguished mature and progenitor melanocytes in human interfollicular epidermis using cKit and CD90 [39], characterization of hair follicle melanocytes has largely relied on anatomical location [40, 41] or differentiation stage [42], lacking functional resolution. Analysis of PSU melanocytes identified four transcriptionally distinct populations (MC1‐MC4) (Figure 5a,b). Among these, MC3 cells expressed *CD63*, *CD81*, *APOE*, and *FTL*, but lacked canonical macrophage markers such as *CD68* and *CD163*, supporting annotation of MC3 as a distinct melanocyte state rather than melanophages.

**FIGURE 5:**
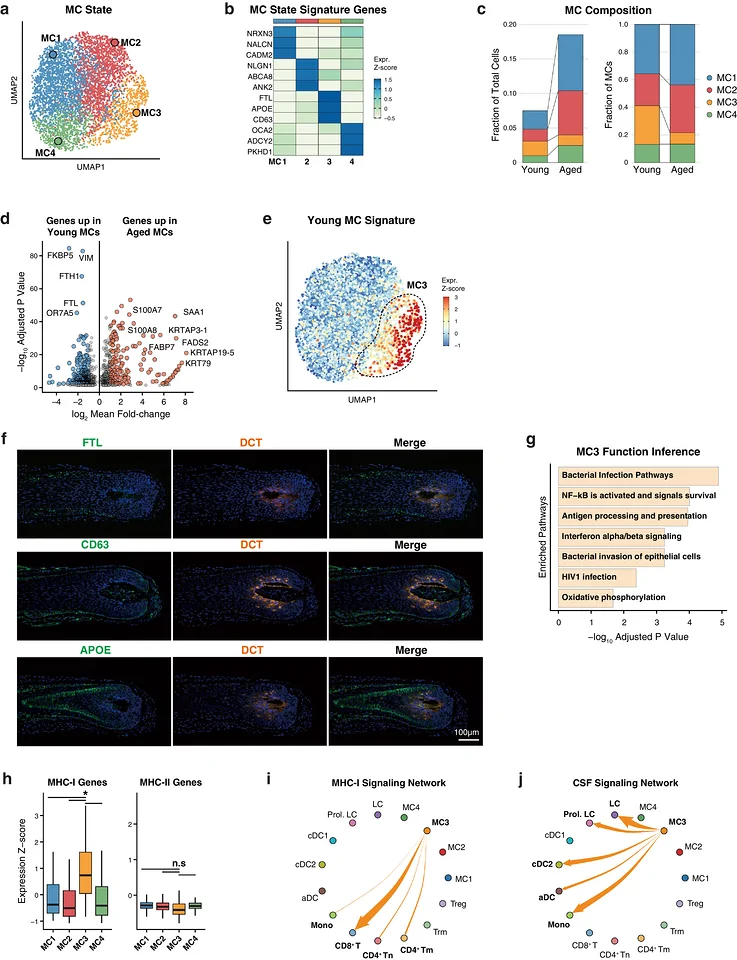
Decline of the innate immune‐like melanocyte subset MC3 with aging. (a) UMAP representation of melanocytes (MCs) resolving four transcriptional states (MC1‐MC4). (b) Heatmap of representative MC state signature genes. (c) Composition analysis comparing relative proportions of MC subpopulations between young and aged PSUs. Left: relative proportion normalized to total cells; right: relative proportion normalized to all MCs. (d) Volcano plot depicting differentially expressed genes between aged and young MCs, highlighting upregulated and downregulated genes. (e) UMAP showing localization of genes enriched in young MCs within the MC3 population. (f) Representative TSA‐based multiplex immunofluorescence images showing DCT (orange), a marker of differentiated bulb melanocytes, the MC3‐associated markers FTL, CD63, and APOE (green), and DAPI (blue); fluorescence channels are shown in pseudocolor. Scale bars, 100 µm. (g) Significantly enriched GO Biological Process terms among MC3 signature genes. (h) Mean expression scores of MHC class I and II molecules across MC subsets. Statistical significance was determined by two‐tailed Wilcoxon rank‐sum test; ^*^: *p* < 0.05; ns: not significant. (i) CellChat‐predicted MHC‐I signaling network from MC3 cells to T‐cell subsets and monocytes in young PSUs. (j) CellChat‐predicted CSF signaling network from MC3 cells to myeloid populations in young PSUs.

During aging, the relative proportion of melanocytes increased within the PSUs, whereas the frequency of the MC3 population markedly declined (Figure 5c). Differential expression analysis revealed age‐associated upregulation of stress‐ and immune‐associated genes, including *SAA1*, *S100A7*, and *S100A8*, together with reduced expression of homeostatic genes such as *FKBP5*, *FTH1*, and *FTL* (Figure 5d). Notably, many genes downregulated in aged melanocytes were preferentially expressed within the MC3 population (Figure 5e), suggesting that reduced representation of this subset may partially contribute to the age‐associated transcriptional changes observed in the melanocyte compartment.

To investigate the biological processes associated with these transcriptional changes, we performed pathway enrichment analyses. Genes downregulated in aged melanocytes were significantly enriched for biological processes related to protein folding, protein stabilization, lysosomal organization, and cellular stress adaptation (Figure S6a), suggesting reduced cellular homeostatic programs. In parallel, gene set enrichment analysis (GSEA) identified enrichment of granulocyte chemotaxis‐related programs in aged melanocytes (Figure S6b), consistent with transcriptional features characteristic of stress and inflammation.

To compare the spatial distribution of MC3‐associated markers with DCT‐positive bulb melanocytes, we performed immunofluorescence co‐staining. DCT signal was concentrated in the hair bulb, whereas FTL, CD63, and APOE signals were detected predominantly outside the DCT‐positive bulb region, with limited spatial overlap (Figure 5f). Functional enrichment analysis of MC3 signature genes revealed enrichment of pathogen defense and inflammatory response pathways, including NF‐κB and IFN‐α/β signaling (Figure 5g). Consistent with these signatures, MC3 cells exhibited elevated major histocompatibility complex (MHC) class I expression relative to other melanocyte states (Figure 5h). Because the MC3 state was markedly reduced in aged PSUs, we characterized MC3‐associated communication in the young dataset, where this state was more highly represented. CellChat predicted MHC‐I‐associated signaling from MC3 cells to T‐cell subsets and monocytes, as well as colony‐stimulating factor (CSF)‐associated signaling from MC3 cells to myeloid populations (Figure 5i,j).

To further evaluate age‐associated alterations in microenvironmental crosstalk, we performed CellChat analysis to infer melanocyte‐to‐myeloid communication networks. Differential network analysis revealed a broad remodeling of melanocyte‐associated communication in aged PSUs, with altered overall interaction patterns between melanocytes and myeloid populations (Figure S6c). However, examination of specific signaling pathways revealed selective reductions in inferred immunomodulatory signaling pathways, including APP‐CD74, GAS6‐AXL/MERTK, IL16, and VEGF signaling (Figure S6d). Together, these findings suggest that age‐associated decline of the MC3 population is accompanied by selective remodeling of melanocyte‐derived communication programs within a broader reorganization of melanocyte–immune interactions during aging.

### 2.6 Age‐Associated Immune Remodeling and Inflammaging in human PSUs

Inflammaging, defined as chronic low‐grade inflammatory changes and immune dysregulation during aging [43], has not been fully characterized in the PSU. To address this, we identified different myeloid and T cell subpopulations. For the myeloid compartment, we identified *CD207* ^+^ *CD1A* ^+^ Langerhans cells (LCs), proliferating LCs, *CD14* ^+^ *FCGR3A* ^+^ (*CD16*) monocytes, conventional dendritic cells (cDC1: *TLR3* ^+^ *IRF8* ^+^; cDC2: *CD1C* ^+^), activated DCs (aDCs, *CD83* ^+^ *CD86* ^+^) (Figure 6a,b). Myeloid cell representation was approximately doubled in aged PSUs, suggesting altered immune composition within the PSU niche (Figure 6c). For the T cell compartment, we identified naïve (*CCR7* ^+^) and memory (*CD95* ^+^) CD4^+^ T cells, *CCL5* ^+^ *GZMK* ^+^ CD8^+^ T cells, *CD69* ^+^ tissue‐resident memory T cells (T_RM_) and *FOXP3* ^+^ *CTLA4* ^+^ regulatory T cells (Tregs), with each population expressing their canonical markers (Figure 6d,e). Similarly, the T cell compartment showed an approximately two‐fold increase in representation in aged PSUs, with the most marked increases observed in T_RM_ and CD8^+^ T cell populations (Figure 6f). Per‐donor quantification verified that aDCs and naïve CD4^+^ T cells proportionally declined with age, whereas T_RM_ cells were proportionally increased, consistent with previously reported age‐associated declines in naïve T cells and expansion of memory T cells [44, 45, 46, 47, 48, 49] (Figure S7a,b).

**FIGURE 6:**
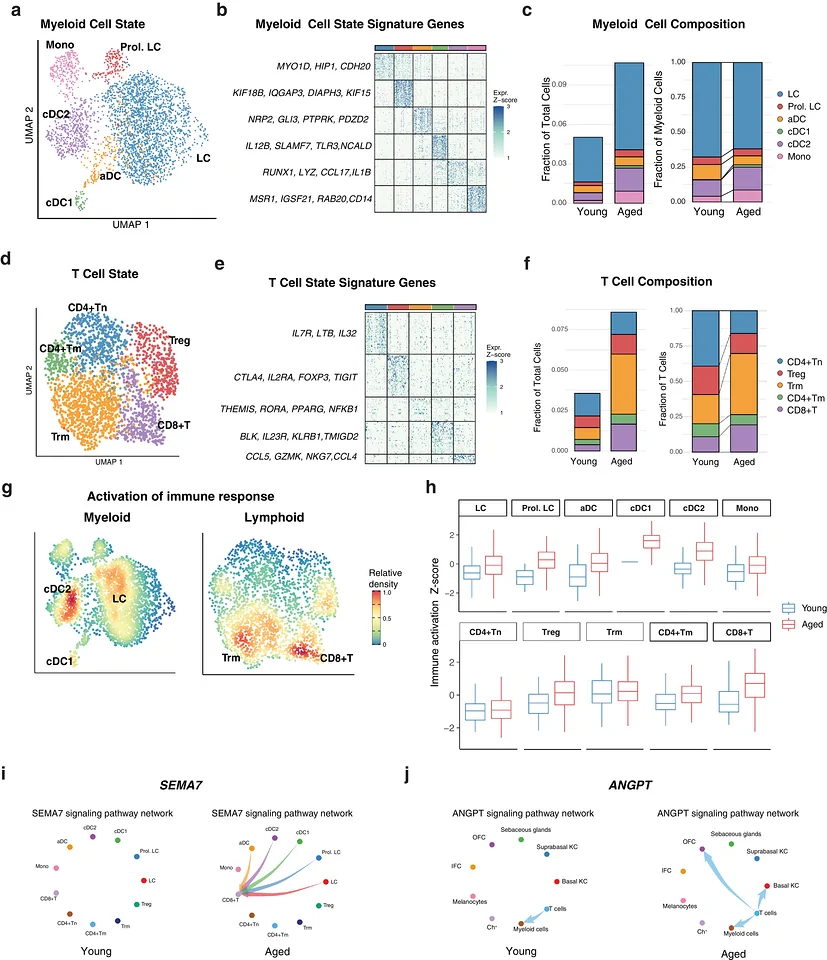
Immune cell composition and intercellular signaling remodeling in aged pilosebaceous units. (a) UMAP representation of myeloid immune cells resolving two Langerhans cell subsets (LC, Proliferating LC), two conventional dendritic cell subsets (cDC1, cDC2), an activated dendritic cell subset (aDC) and a monocyte population (Mono). (b) Heatmap of myeloid cell state signature genes (scaled Z‐scores) with representative marker genes listed on the left; each column corresponds to a state shown in (a). (c) Composition analysis comparing relative proportions of myeloid subpopulations between young and aged PSUs. Left: relative abundance normalized to total cells; right: relative abundance normalized to all myeloid cells. (d) UMAP representation of T cells resolving five transcriptional subsets. (e) Heatmap of T‐cell state signature genes (scaled Z‐scores) with representative marker genes listed on the left; each column corresponds to a state shown in (d). (f) Composition analysis comparing relative proportions of T‐cell subpopulations between young and aged PSUs. Left: relative abundance normalized to total cells; right: relative abundance normalized to all T cells. (g) Nebulosa density plots showing activation of immune response gene modules in myeloid (left) and T‐cell (right) populations. (h) Box plot showing expression z‐scores of immune activation gene modules. (i) Predicted cell‐cell communication network showing ligand‐receptor interactions from myeloid subsets to CD8^+^ T cells through SEMA7 signaling in young (left) and aged (right) samples. (j) Predicted ligand‐receptor interactions between T cells and other PSU cell types through ANGPT signaling in young (left) and aged (right) samples.

Differential gene expression and pathway enrichment analyses revealed broadly enhanced immune activation in both myeloid and T cell compartments (Figure S7c). This functional gene set expression was particularly high in cDC1, cDC2, LCs, CD8^+^ T cells, and T_RM_ (Figure 6g). With the exception of T_RM_, cDC1, cDC2, LCs, and CD8^+^ T cells all exhibited heightened immune response scores in aged PSU, suggesting that these populations may contribute to inflammaging‐associated immune remodeling within the PSU microenvironment (Figure 6h). Cell‐cell interaction analysis suggested increased inferred intercellular communication between myeloid cells and T cells in aged PSUs, particularly between cDC1s and Tregs (Figure S8a,b), which may reflect altered immune regulatory interactions involved in balancing immune activation and tolerance during aging [14, 50]. Beyond this, CellChat analysis revealed global remodeling of intercellular communication in aged PSU. At the immune cell subset level, DCs and LCs exhibited significantly increased *SEMA7A*‐mediated signaling toward CD8^+^ T cells, consistent with enhanced immune surveillance (Figure 6i; Figure S8c). Considering other cell types in PSUs, T cells exhibited increased inferred *ANGPT* and *CD40* signaling toward OFCs and BKCs, whereas inferred *IL10*‐mediated interactions with channel^+^ cells were reduced (Figure 6j; Figure S9a,b). Collectively, these findings indicate extensive remodeling of immune–immune and immune–epithelial communication networks in aged PSUs, characterized by increased inferred pro‐inflammatory and angiogenic signaling together with reduced immunoregulatory interactions.

Metabolic pathway analysis further revealed age‐associated metabolic alterations: branched‐chain amino acid (BCAA) synthesis and metabolism were elevated in myeloid cells (Figure S9c), consistent with previous studies linking BCAA metabolism to senescence‐associated secretory phenotypes (SASP) [51], while tryptophan catabolism, a pathway implicated in immune regulation [52], was enhanced in cDC1s (Figure S9d). Overall, these findings demonstrate that aged PSUs undergo coordinated immune and metabolic remodeling, characterized by increased immune cell representation, altered intercellular communication, and metabolic adaptations that may collectively contribute to local inflammaging.

## 3 Discussion

Although vellus hair lacks the conspicuous growth dynamics characteristic of terminal hair follicles, its associated PSU represents a critical yet understudied component of human skin biology. Distributed across most body surfaces, vellus PSUs form a widespread epithelial–immune–sebaceous network that contributes to barrier integrity, lipid homeostasis, tissue repair, and local immune regulation [53]. Consequently, age‐associated dysfunction of this niche may have implications extending beyond hair biology, potentially contributing to clinically relevant skin‐aging phenotypes including xerosis [54], impaired wound healing, microbial imbalance, and chronic low‐grade inflammation [55]. Despite their ubiquity and physiological importance, the cellular and molecular mechanisms underlying vellus PSU aging have remained poorly understood.

To address this gap, we generated the first comparative single‐nucleus transcriptomic atlas of human vellus hair PSUs from young and aged individuals. By systematically resolving 12 major cell populations and more than 30 transcriptionally distinct cellular states, we characterized age‐associated remodeling across epithelial, sebaceous, melanocytic, and immune compartments. Beyond defining changes in cellular composition, our analyses uncovered previously unrecognized alterations in epithelial state organization, transcriptional regulatory programs, metabolic states, and intercellular communication networks. Together, these findings establish a comprehensive cellular framework for understanding how aging reshapes the human vellus PSU microenvironment.

Among PSU compartments, age‐associated transcriptomic alterations were particularly prominent within bulge‐associated lineages. The age‐related decline of bulge HFSC representation was accompanied by transcriptional features consistent with reduced regeneration‐associated programs, suggesting compromised regenerative homeostasis within follicular compartments [56]. This vulnerability likely reflects the cumulative impact of intrinsic and extrinsic stressors, including DNA damage, altered niche signaling, and chronic inflammation. In parallel, multiple metabolic pathways exhibited age‐associated remodeling, including increased activity of sialic acid metabolism, glycosaminoglycan biosynthesis, and biotin metabolism. Collectively, these observations identify the bulge niche as an important site of age‐associated remodeling within human vellus PSUs.

Sebaceous glands are increasingly recognized as active regulators of skin homeostasis rather than passive lipid‐producing structures. SG holocrine secretion has long been described through morphologically defined zones [57], yet by resolving the sebocyte differentiation continuum at single‐cell resolution, our atlas provides a molecular framework linking proliferative basal sebocytes to terminal secretory cells. In addition to refining classical histological zonation, these data highlight stage‐specific metabolic programs associated with sebocyte maturation. Notably, aging was accompanied by transcriptional remodeling of sebaceous gland cells, most prominently reflected by increased *AR*‐associated activity. Because sebaceous gland activity is tightly regulated by endocrine signaling, enhanced *AR* activity may represent a transcriptional adaptation associated with increased lipogenic programs despite age‐associated cellular stress and declining metabolic efficiency. Alternatively, increased androgen responsiveness could contribute to age‐related alterations in sebum composition, potentially affecting epidermal barrier integrity and local inflammatory responses. Given the central role of sebum in regulating microbial ecology, skin hydration, and immune homeostasis, these findings further support the concept that age‐associated alterations in sebaceous gland transcriptional states may represent an important contributor to broader PSU niche remodeling during skin aging.

A notable finding of this study was the identification of an age‐associated channel^+^ epithelial state that displayed reproducible enrichment across independent aging and inflammatory skin datasets. Multiple lines of evidence support the interpretation that channel^+^ cells represent a stress‐responsive epithelial state rather than a previously defined canonical lineage. Trajectory analysis positioned channel^+^ cells as a blind‐ended branch within the outer follicular epithelial trajectory, while CytoTRACE suggested an intermediate differentiation state. Concurrently, SCENIC analysis revealed age‐associated remodeling of transcriptional regulatory programs in channel^+^ cells, characterized by reduced activity of epithelial homeostasis‐associated regulons and increased activity of stress‐responsive and inflammatory regulons, including *FOSL2*, *STAT3*, and *IRF1*.

Together, these findings suggest that channel^+^ cells represent a specialized epithelial state associated with epithelial remodeling within the inner follicular compartment. Consistent with this interpretation, aging was accompanied by substantial remodeling of inferred communication networks involving channel^+^ cells, characterized by reduced homeostatic trophic communication and enhanced stress‐associated signaling programs. In particular, the shift from *FGF7*‐associated signaling toward *NAMPT*‐associated signaling, together with the positive correlation between *FOSL2* activity and *NAMPT* expression, is consistent with a potential role for channel^+^ cells in shaping stress‐associated molecular interactions within the aging PSU microenvironment.

Although the present study does not establish a causal role for channel^+^ cells in tissue aging or inflammatory skin disease, their reproducible enrichment across aging and psoriasis datasets, together with their stress‐responsive transcriptional and signaling features, suggests that they may represent a shared epithelial response to chronic tissue stress. Future studies integrating spatial transcriptomics and functional perturbation approaches will be required to determine whether channel^+^ cells actively contribute to niche remodeling or primarily serve as indicators of epithelial adaptation during aging.

We identified marked heterogeneity within the melanocyte compartment of human vellus PSUs, including a distinct subset (MC3) characterized by immune‐associated transcriptional features. Spatial mapping showed that DCT^+^ melanocytes are predominantly localized within the hair bulb, whereas MC3‐associated markers (*FTL*, *CD63*, and *APOE*) were detected mainly outside the DCT^+^ bulb region, displaying limited spatial overlap with DCT^+^ cells. Importantly, the absence of macrophage‐specific transcripts such as *CD68* supports the interpretation that MC3 cells represent a melanocyte‐intrinsic state rather than contaminating melanophages. A direct correspondence between MC3 cells and previously reported ORS‐resident amelanotic melanocytes [58, 59] remains to be established. Notably, MC3 cells exhibited enrichment of pathogen defense and inflammatory response pathways, and elevated MHC class I expression; in young PSUs, CellChat further predicted MC3‐associated communication with T‐cell and myeloid populations, consistent with emerging evidence that melanocytes contribute to immune regulation within the skin niche in addition to their canonical role in pigmentation [60].

Aging was associated with a reduced relative frequency of the MC3 state, and selective reductions in multiple inferred melanocyte‐derived communication pathways. Although global melanocyte–immune interaction networks underwent broader remodeling during aging, several immunomodulatory signaling programs, including *APP*‐*CD74*, *GAS6*‐*AXL*/*MERTK*, *IL16*, and *VEGF* signaling, were preferentially diminished. These observations suggest that age‐associated decline of MC3 is accompanied by remodeling of specific melanocyte‐associated communication programs within the PSU niche. Given the descriptive nature of single‐nucleus transcriptomic analyses, however, we refrain from inferring direct functional consequences or causal roles in tissue aging. Future spatial multi‐omics and functional studies will be required to determine whether MC3 represents a stable melanocyte subtype, a reversible activation state, or a context‐dependent adaptive phenotype.

Collectively, increased representation of a stress‐responsive channel^+^ epithelial state, the decline of immune‐associated MC3 melanocytes, and the remodeling of resident immune populations suggest that aging affects the PSU as an integrated niche rather than as isolated cellular compartments. These coordinated changes suggest a transition in the balance between tissue maintenance programs, stress adaptation, and inflammatory signaling during aging, providing a potential cellular framework for cutaneous inflammaging.

Beyond epithelial and melanocyte remodeling, aging also reshaped the immune landscape of the PSU niche. The preferential accumulation of immune cells within aged PSUs is consistent with the concept of cutaneous inflammaging, in which sustained immune cell accumulation accompanies age‐associated epithelial and niche remodeling. In aged PSUs, immune composition and transcriptional programs collectively exhibit changes consistent with a chronic low‐grade inflammatory state, accompanied by alterations related to immune aging, metabolic remodeling, and cellular stress responses. We observed hallmarks associated with immunosenescence, including reduced representation of aDCs, alongside the proportional expansion of memory T cell populations. In parallel, myeloid cells exhibited metabolic and transcriptional features previously associated with senescence‐associated secretory phenotypes, while cDC1s showed altered expression of tryptophan metabolism‐related genes, suggesting changes in immunoregulatory balance. Together, these observations indicate that immune remodeling in aged PSUs is accompanied by coordinated transcriptional and metabolic changes, contributing to a locally inflamed microenvironment characteristic of skin aging.

Our study has several limitations. First, nuclei were isolated from epidermis‐enriched PSUs, which may underrepresent deeper dermal niche cells. Age‐associated differences in extracellular matrix composition and tissue properties may also affect PSU recovery and nuclei isolation; despite consistent processing across age groups, differential recovery of cellular populations cannot be fully excluded. Second, individual cumulative ultraviolet exposure was not quantified for the back‐skin samples, and residual photoexposure‐related variation may therefore influence the interpretation of age‐associated melanocyte changes. Third, the sample size, exclusive use of male donors, and pooling of aged nuclei limit the assessment of sex‐specific and inter‐individual variability. Finally, metabolic pathway activities were inferred from nuclear transcriptomic signatures and should therefore be interpreted as transcriptional potential rather than direct measures of metabolic flux. Future studies incorporating larger, sex‐balanced cohorts and functional validation will be required to extend these findings.

In summary, we present the first single‐nucleus transcriptomic atlas of human vellus hair‐associated PSUs across age groups, establishing a comprehensive cellular and molecular framework for vellus PSU aging. By uncovering coordinated remodeling across epithelial, sebaceous, melanocytic, and immune compartments, our study identifies PSU niche reorganization as a prominent feature of aging skin and provides a valuable resource for investigating how aging reshapes the widespread vellus PSU network and influences cutaneous homeostasis, inflammaging, and age‐associated skin dysfunction.

## 4 Methods

### 4.1 Sample Collection and Preparation

Human back skin samples (approximately 1 cm^3^) were obtained as non‐lesional surgical margins from male donors (three young, 20–40 years; six aged, 60–93 years) undergoing minor surgical procedures at Huashan Hospital. Back skin was selected because the study focused on intact vellus hair PSUs, including the sebaceous gland compartment, and this site provided sufficient PSUs for isolation and transcriptomic profiling. This all‐male cohort design was strategically chosen to minimize confounding effects of sex‐specific hormonal variations on pilosebaceous unit biology, particularly given the known influence of androgens on sebaceous gland function. For aged donors, to ensure sufficient nuclear yield and transcriptomic coverage from the small‐sized vellus hair follicles while preserving high‐quality cell‐type‐specific transcriptional signatures, tissues from two or three individuals were pooled to obtain three representative aged samples for library preparation. The tissue was rinsed with phosphate‐buffered saline (PBS), and subcutaneous adipose tissue was removed. Fresh skin specimens were incubated in 2.5 mg/mL Dispase II (Roche) in Dulbecco's modified Eagle medium (DMEM) overnight at 4°C to separate the epidermis from the dermis. The epidermal pilosebaceous units were then manually separated from the interfollicular epidermis under a stereomicroscope. Samples from young donors were divided into two equal portions for parallel processing by single‐cell dissociation and nuclei isolation. Aged donor samples were processed exclusively for nuclei isolation. The study was approved by the Institutional Review Board of Huashan Hospital, Fudan University (KY2024‐098) and written informed consent was obtained from all participants.

### 4.2 Single‐Cell Isolation and Sequencing

Epidermal PSUs were dissociated into single cells by incubation with trypsin at 37°C for 15 min. The resulting cell suspension was filtered through a 70 µm cell strainer, centrifuged at 500 × g for 5 min, and washed with 1× Dulbecco's phosphate‐buffered saline (DPBS) containing 2% fetal bovine serum (FBS). Cell viability was assessed using 0.4% Trypan Blue and a Countess II Automated Cell Counter. The single‐cell suspensions were immediately loaded onto a Chromium Single Cell Controller (10x Genomics) for droplet‐based barcoding using the Chromium Single Cell 3′ Library and Gel Bead Kit v3 (10x Genomics). Sequencing libraries were prepared according to the manufacturer's protocol and sequenced on an Illumina NovaSeq 6000 platform.

### 4.3 Single‐Nucleus Isolation and Sequencing

Nuclei isolation was performed as previously described [61]. Briefly, tissues were homogenized in lysis buffer from the Cell Nuclear Isolation Kit (Shbio, #52009‐10, Shanghai, China) and incubated on ice for 10 min. The lysate was filtered through a 40 µm strainer, centrifuged at 500 × g for 5 min at 4°C, and the pellet was resuspended in lysis buffer. Debris was removed by density gradient centrifugation using the kit's separation solution. Nuclei were washed three times in wash/resuspension buffer, stained with DAPI (10 µg/mL), and counted using a Countess II Automated Cell Counter (Thermo Fisher Scientific). Nuclei were immediately loaded onto a Chromium Single Cell Processor (10x Genomics) for library preparation using the Chromium Single Cell 3′ Library and Gel Bead Kit v3 (10x Genomics), and sequenced on a NovaSeq 6000 system (Illumina).

### 4.4 Single‐Cell and Single‐Nucleus RNA‐seq Data Analysis

#### 4.4.1 Gene Expression Quantification and Integration

All scRNA‐seq and snRNA‐seq data generated in this study were processed using a standardized pipeline adapted from our previous studies [62]. Raw sequencing reads were aligned to the human reference genome (GRCh38) using the 10x Genomics CellRanger toolkit (v7.0.1, cellranger count) with the parameter “–include‐introns = true”; all other settings were left at default. Quality control metrics produced by CellRanger were examined for each library. To remove background signals arising from ambient RNA, raw unique molecular identifier (UMI) count matrices were further processed with CellBender (v0.1.0, remove‐background), using the following parameters: “–total‐droplets‐included = 25 000,” “–low‐count‐threshold = 15,” and “–epochs = 200”. To minimize the loss of valid nuclei, the “–expected‐cells” parameter was set to 1.5 times the number of nuclei reported by CellRanger. Only barcodes identified as valid by both CellRanger and CellBender were retained for downstream analysis. Additional nucleus‐level quality control was performed separately for each library based on UMI counts, detected gene counts, and mitochondrial UMI fractions. Low‐quality nuclei and distributional outliers were removed using library‐specific filtering thresholds. Nuclei passing quality control were screened for doublets using Scrublet (v0.2.3) with parameters expected_doublet_rate = 0.15 and call_doublets_threshold = 0.25. To avoid over‐filtering, nuclei flagged as potential doublets were retained for clustering and further annotation. Finally, all libraries were integrated and batch effects corrected using scVI, a probabilistic modeling framework provided by the scvi‐tools suite (v1.3.3).

#### 4.4.2 snRNA‐seq Library Demultiplexing

Because of limited biopsy material, sample multiplexing was applied for two snRNA‐seq libraries from aged donors: one library pooled nuclei from two donors and the other pooled nuclei from three donors. To recover donor‐specific information, computational demultiplexing was performed using Freemuxlet (v0.1), which infers the genotype of each nucleus in pooled libraries as previously described [63]. As a reference, we used common biallelic single‐nucleotide variants (SNVs; release 20190312) from the 1000 Genomes Project (ftp.1000genomes.ebi.ac.uk). A total of 6,826,029 common SNVs were retained and used as input for popscle dsc‐pileup and popscle freemuxlet to assign nuclei to their respective donors.

#### 4.4.3 Clustering and Annotation

Expression matrix was log‐normalized using Seurat (v5.2.1). We constructed a shared nearest‐neighbor graph using 50 dimensions of the scVI latent space with the FindNeighbors function in Seurat. Clusters were defined on this graph using the Louvain algorithm with an optimized resolution of 1.0. Major cell clusters were annotated based on the reference human skin single‐cell atlas reported by Eraslan et al. [25]. Cell type identities were predicted using the SingleR algorithm, with the original annotations from the skin atlas serving as the reference. Each major cell type was then re‐clustered iteratively: within each cell type, we rebuilt the nearest‐neighbor graph from the full scVI latent dimensions and applied FindClusters at resolutions chosen to achieve clear Uniform Manifold Approximation and Projection (UMAP) separation, robust detection of >30 significantly differentially expressed genes per subcluster, and a minimum area under the curve (AUC) >0.8, as assessed by the Single‐Cell Clustering Assessment Framework (SCCAF). Subclusters enriched for Scrublet‐predicted doublets were further examined for cell type marker gene and lineage‐specific gene expression, and ambiguous profiles were excluded. After quality control, doublet assessment, integration, and annotation, the final dataset comprised 40,145 single‐cell and single‐nucleus profiles with unambiguous annotations for downstream analyses.

#### 4.4.4 Public Dataset Reanalysis

Published human transcriptomic datasets from Eraslan et al. [25] and Cheng et al. [37] were reanalyzed for cross‐dataset validation of the channel^+^ epithelial state. Data associated with the Eraslan et al. study are available through the Broad Institute Single Cell Portal (SCP1479), whereas sequence data from the Cheng et al. study are deposited in the European Genome‐phenome Archive (EGA; EGAS00001002927). These published datasets were reanalyzed for the cell‐state and signature comparisons presented in Figure 4 and Figure S5.

#### 4.4.5 Pseudobulk Principal Component Analysis

To generate pseudobulk profiles, raw UMI counts for the top 5,000 highly variable genes were summed across all cells within each sample. The set of highly variable genes was identified using the FindVariableFeatures function in Seurat with default parameters. Pseudobulk expression matrices were constructed using the AggregateExpression function, followed by normalization. Principal component analysis (PCA) was then performed on the normalized pseudobulk matrix using DESeq2 (v1.42.1).

#### 4.4.6 Detection of Differentially Expressed Genes

Differentially expressed genes (DEGs) were identified using the FindAllMarkers function in Seurat with the Wilcoxon rank‐sum test to define cluster‐enriched signatures. Only upregulated genes were reported, applying a minimum log2 fold‐change threshold of 0.25 and an adjusted p‐value < 0.05 (Benjamini‐Hochberg correction).

#### 4.4.7 Gene Set Expression Scoring

Gene set scoring was performed using the AddModuleScore function in Seurat, which computes a per‐cell expression score for a given gene set. For each cell, the score is defined as the mean expression of genes in the specified gene set minus the mean expression of a background gene set. Background genes are randomly sampled (100 genes per query gene) from those with similar mean expression levels across all cells, thereby controlling for expression bias. Resulting scores were scaled and centered to produce Z‐scores. Comparisons of gene module scores between sample groups were conducted using the non‐parametric two‐tailed Wilcoxon rank‐sum test. For aging signature analysis, we used the GTEx_Aging_Signatures_2021 database. In this resource, 479 “young skin” signature genes were defined as those significantly upregulated in skin samples from 20–29‐year‐old donors compared with 60–79‐year‐old donors. Conversely, 395 “aged skin” signature genes were defined as those significantly downregulated in the same comparison.

#### 4.4.8 Functional Enrichment and Gene Set Enrichment Analysis (GSEA)

Functional enrichment analyses, including Gene Ontology (GO) over‐representation analysis and GSEA, were performed using the clusterProfiler R package (v4.10.1). GO enrichment was conducted using the org.Hs.eg.db database (v3.18.0) within the Biological Process (BP) ontology. For GSEA, genes were ranked based on the average log2 fold change from differential expression analysis, and enrichment was evaluated against the Hallmark gene sets from the Molecular Signatures Database (MSigDB). Statistical significance was determined using the Benjamini‐Hochberg method to control the false discovery rate (FDR), with adjusted p‐value < 0.05 considered significant.

#### 4.4.9 Differential Abundance Testing

Differential abundance testing was performed using MiloR (v1.10.0) to compare cell‐type frequencies between conditions. A k‐nearest neighbor graph was constructed from the 50‐dimensional scVI latent space, with the number of nearest neighbors (k) set to 20 and the proportion of randomly sampled vertices (prop) set to 0.5. Statistical significance was assessed using a quasi‐likelihood F‐test with an α threshold of 0.1, and *p*‐values were corrected for multiple testing using the Benjamini‐Hochberg method.

#### 4.4.10 Trajectory and Pseudotime Inference

Pseudotemporal trajectory inference was performed using the Slingshot R package (v2.8.0). Trajectories were constructed based on the top 50 scVI latent dimensions, and visualized on UMAP embeddings. Pseudotime values were inferred along the resulting trajectories and used for downstream analyses.

#### 4.4.11 CytoTRACE Analysis

CytoTRACE analysis was performed using the CytoTRACE R package (v0.3.3) to infer relative differentiation states of epithelial populations. Normalized gene expression matrices from Seurat were used as input. CytoTRACE scores were computed based on transcriptional diversity and gene count per cell, where higher scores indicate less differentiated states.

#### 4.4.12 SCENIC Analysis

Single‐Cell Regulatory Network Inference and Clustering (SCENIC) analysis was performed using pySCENIC (v0.12.1) to infer transcription factor regulatory network activity at single‐cell resolution. Gene regulatory networks were inferred based on transcription factor‐target co‐expression, followed by motif enrichment to define regulons. Regulon activity scores were calculated using AUCell and visualized across cell populations using heatmaps and UMAP embeddings.

#### 4.4.13 Cell–Cell Communication Inference

Cell–cell communication analysis was performed using CellChat (v1.5.0) on the snRNA‐seq datasets, with ligand‐receptor interactions and communication networks inferred using the standard CellChat workflow and the human ligand‐receptor database (CellChatDB.human). For analyses comparing young and aged conditions, communication networks were inferred independently within each age group using consistent cell‐type or cell‐state annotations and identical analysis settings within each comparison. The resulting age‐specific CellChat objects were subsequently merged only for between‐group comparison and visualization. The descriptive analyses of MC3‐associated communication shown in Figure 5i,j were performed using the young snRNA‐seq dataset and were restricted to melanocyte, myeloid, and T‐cell populations annotated at the cell‐state level.

#### 4.4.14 Metabolic Program Inference

Metabolic pathway activity was inferred at single‐cell resolution using scMetabolism, an R‐based tool for quantifying metabolism‐related gene set activity. Briefly, raw count matrices were used as input, and pathway activity scores were calculated based on curated Kyoto Encyclopedia of Genes and Genomes (KEGG) metabolic gene sets provided in the scMetabolism package. Activity scores for each pathway were computed by aggregating expression levels of pathway‐associated genes, normalized by library size, and scaled across cells. The resulting scores reflect relative pathway activity across cell types or states. For visualization, pathway activity matrices were integrated with the single‐cell metadata to enable comparison across defined clusters, pseudotime trajectories, and experimental conditions.

#### 4.4.15 Statistical Analysis

Statistical analyses were performed using R (v4.3.2). For comparisons of gene expression levels, pathway or signature scores, and cell‐type proportions between two groups, two‐tailed Wilcoxon rank‐sum tests or Student's *t*‐tests were applied as indicated in the figure legends, depending on data distribution. To assess differences in global or lineage‐specific cellular distributions along pseudotime trajectories, the two‐sided Kolmogorov‐Smirnov (KS) test was utilized. In box plots, the center line represents the median, box limits represent the upper and lower quartiles, and whiskers represent 1.5× interquartile range (IQR). *P*‐values were adjusted for multiple comparisons using the Benjamini‐Hochberg method where applicable. Unless otherwise stated, statistical significance is denoted as follows: ^*^: *p* < 0.05, ^**^: *p* < 0.01, ^***^: *p* < 0.001, and ^****^: *p* < 0.0001; ns: not significant.

### 4.5 Immunohistochemistry Staining

Human skin tissue samples were fixed in 4% paraformaldehyde and embedded in paraffin. Sections were deparaffinized, rehydrated, and subjected to antigen retrieval using EDTA buffer (pH 9.0) at 100°C for 23 min. After cooling, sections were washed three times in PBS and incubated in 3% H_2_O_2_ for 15 min at room temperature. After blocking with 5% bovine serum albumin (BSA) for 30 min, sections were incubated with a primary antibody against LEF1 (Abcam, 1:200) at 4°C overnight. After three washes in PBS containing Tween‐20 (PBST), the horseradish peroxidase (HRP)‐conjugated secondary antibody was used before the 3,3′‐diaminobenzidine (DAB) application. Sections were counterstained with hematoxylin, dehydrated sequentially in graded ethanol (75%, 85%, 100%, and 100%) and xylene, and mounted with resinous medium.

### 4.6 Immunofluorescence Staining

Paraffin‐embedded skin sections were subjected to antigen retrieval using EDTA buffer (pH 9.0) at 100°C for 23 min, followed by cooling to room temperature and washing three times in PBS. After blocking with 5% BSA for 1 h at room temperature, sections were incubated in primary antibodies at 4°C overnight. For conventional immunofluorescence staining, sections were washed three times in PBST and incubated with appropriate secondary antibodies conjugated with Alexa Fluor 488 or 647 (Abcam). For co‐staining of DCT with CD63, FTL, or APOE, tyramide signal amplification (TSA)‐based multiplex immunofluorescence was performed using a TSA kit (Lianlanbio Biological Technology, LL20013, Shanghai, China) according to the manufacturer's protocol. DAPI was used for nuclear counterstaining. Images were acquired using the Zeiss Cell Observer System (Zeiss). Fluorescence channels in Figure 5f were pseudocolored for visualization. The antibody dilutions used were as follows: FASN (rabbit, Proteintech, 1:200), Ki‐67 (mouse, CST, 1:400), FASN (mouse, Proteintech, 1:400), CD44 (rabbit, Proteintech, 1:200), AACS (rabbit, Proteintech, 1:100), PLIN5 (rabbit, Proteintech, 1:200), AR (mouse, Proteintech, 1:100), TG (mouse, Proteintech, 1:100), EYA2 (rabbit, Proteintech, 1:50), DCT (rabbit, Abcam, 1:300), CD63 (rabbit, Proteintech, 1:1000), FTL (rabbit, Abcam, 1:100), and APOE (rabbit, Abcam, 1:500).

## Author Contributions

**Wei Li** and **Xu Yao** conceptualized the project and supervised the work. **Zhuoqiong Qiu** collected biopsies from donors undergoing minor surgical procedures. **Ya'nan Li** and **Zhuoqiong Qiu** performed isolation of skin PSU cells and nuclei. **Ya'nan Li**, **Xiaoyu Pan**, and **Geyang Xu** performed bioinformatics analysis of the scRNA‐seq and snRNA‐seq data. **Ya'nan Li**, **Zhuoqiong Qiu**, **Xiaoyu Pan**, **Geyang Xu**, **Shanshan Peng**, **Ronghui Zhu**, **Qiuyang Luo**, and **Xiaokai Fang** performed the validation experiments and interpreted the data. **Ya'nan Li** wrote the manuscript, and all authors revised and approved the final draft.

## Conflicts of Interest

The authors declare no conflicts of interest.

## Supporting information




**Supporting File 1**: advs78048‐sup‐0001‐SuppMat.pdf.

**Supporting File 2**: advs78048‐sup‐0002‐TableS1.xlsx.

## Data Availability

The data that support the findings of this study are openly available in GSA‐Human at https://ngdc.cncb.ac.cn/gsa‐human, reference number HRA013875.
