# Supplementary material for: Single‐Nucleus Transcriptomic Atlas of Human Vellus Hair Pilosebaceous Units Reveals Age‐Associated Remodeling
Source: Adv Sci (Weinh). 2026 Sep 29:e78048. Online ahead of print. doi: 10.1002/advs.78048 (PMC13624415; doi:10.1002/advs.78048)
Supplement: Supplementary file 1 [file ADVS-9999-e78048-s002.pdf]

Supplemental Information

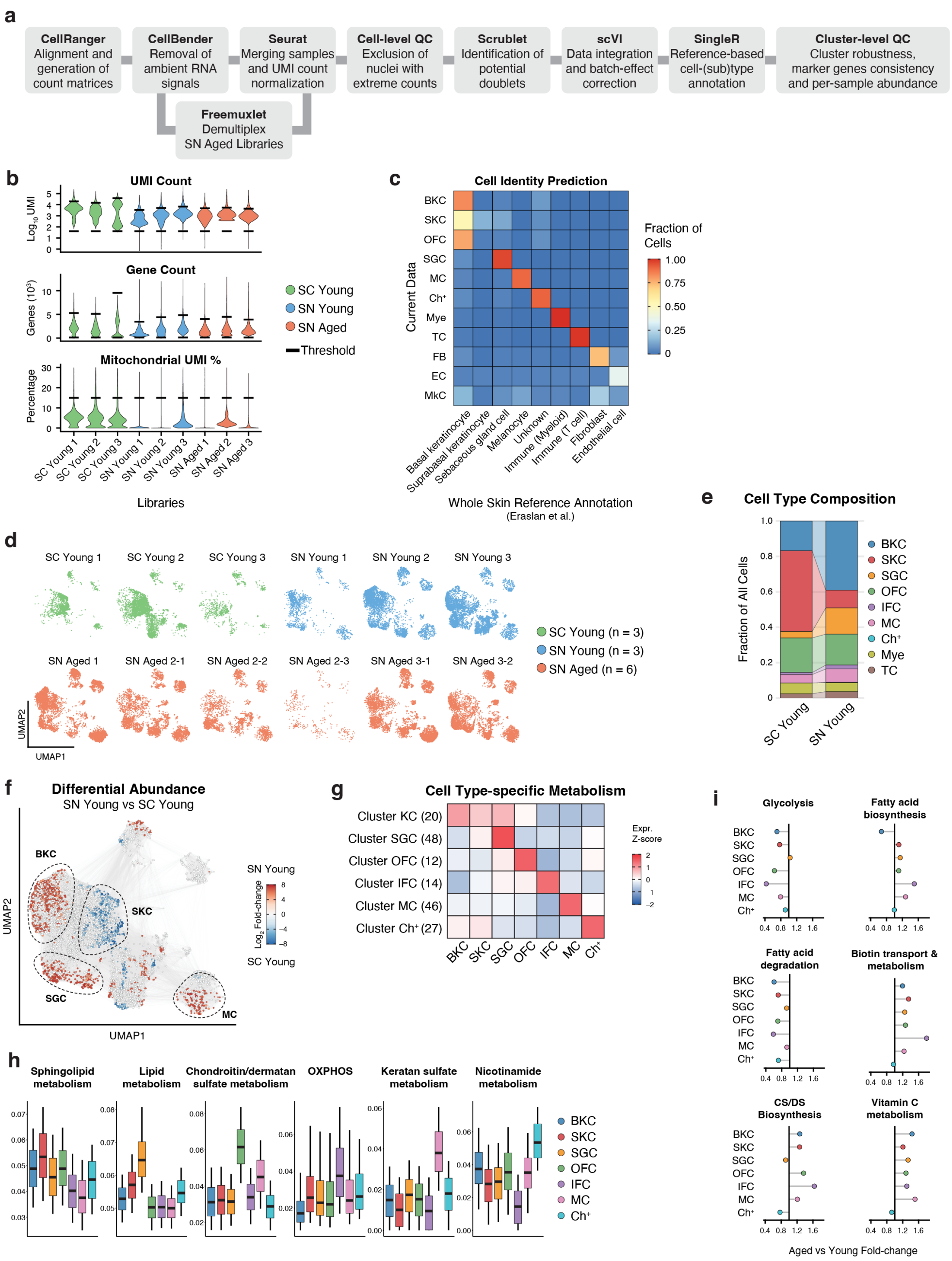

Figure S1. Processing and integration of sn/scRNA-seq data from human pilosebaceous units.

a, Schematic showing key steps in the sn/scRNA-seq data processing pipeline. **b**, Violin plots showing per-library quantification of UMI counts, detected gene counts, and mitochondrial UMI percentages. Black bars represent per-library filtering cutoffs for outlier removal. **c**, Heatmap showing reference-assisted annotation of cell clusters in the integrated dataset. Each row represents a cluster in the current dataset, and each column represents a reference cell type

in the human skin atlas. Note that Channel^{+} cells correspond to cells annotated as “Unknown” in the reference atlas. **d**, UMAP representation showing all cells split by library. **e**, Composition of major cell types identified in single-nucleus and single-cell RNA-seq data from young skin samples. **f**, Milo differential abundance analysis showing populations enriched in single-cell versus single-nucleus datasets. **g**, scMetabolism-inferred clustering of metabolic pathways enriched in each major cell type. Numbers indicate pathway counts per cluster. **h**, Box plot showing expression scores of genes from a representative pathway in each metabolic cluster (**g**) across cell types. **i**, Differential metabolic pathways between age groups across PSU cell types.

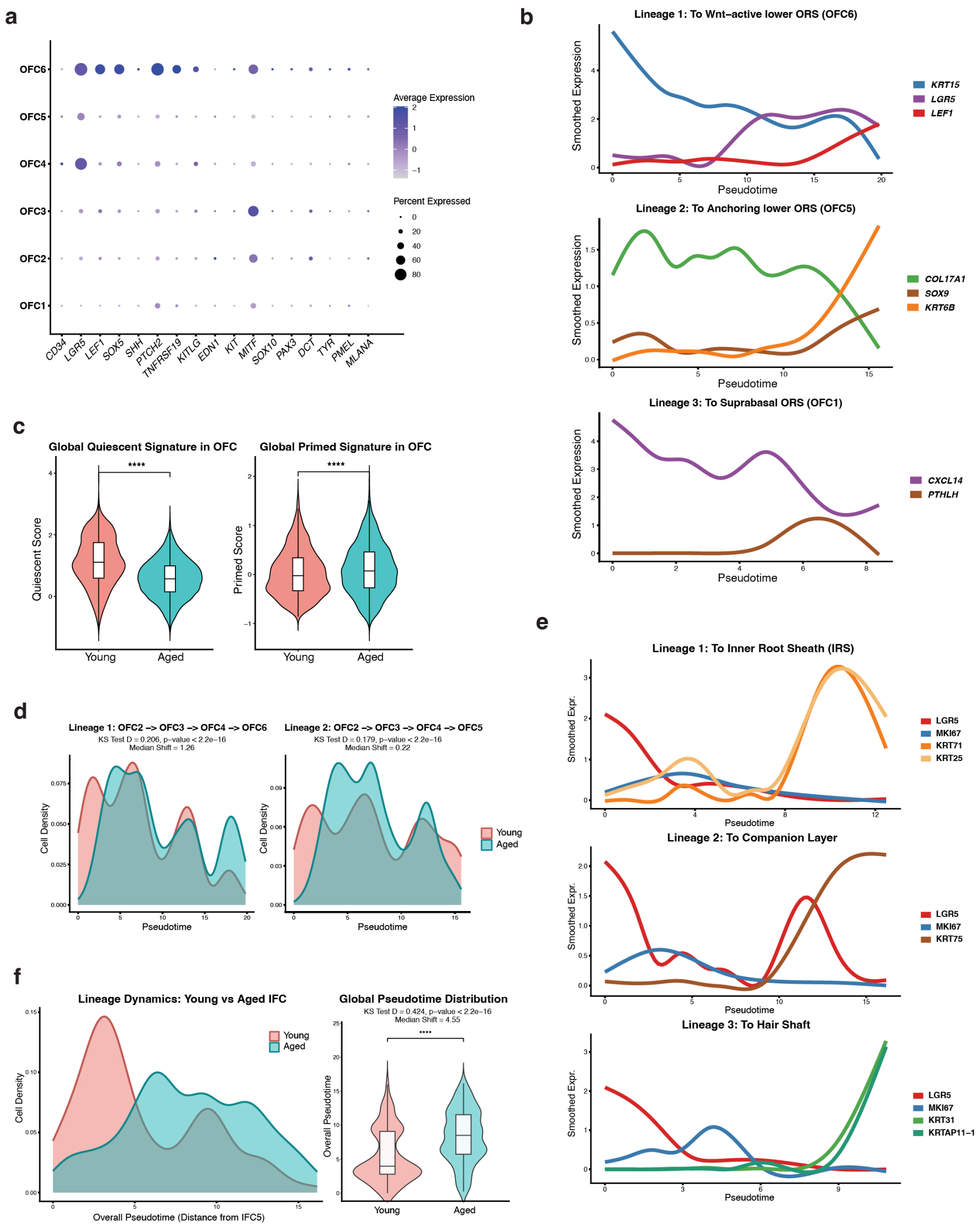

Figure S2. Trajectory inference and progenitor-state characterization of follicular epithelial compartments.

a, Characterization of the OFC6 state showing expression of Wnt/Shh-associated genes, epithelial stem/progenitor markers, together with limited expression of melanocyte-lineage markers. **b,** Expression dynamics of representative markers along OFC pseudotime trajectories modeling progression toward OFC1, OFC5, and OFC6 states. **c,** Quiescent HFSC and primed progenitor signature scores in OFCs stratified by age (****: $p < 0.0001$, Wilcoxon rank-sum

test). **d,** Density distribution of young and aged OFC cells along lineage-specific OFC pseudotime trajectories. Statistical significance for density shifts was assessed via the two-sided Kolmogorov-Smirnov (KS) test. **e,** Expression dynamics of representative markers along IFC pseudotime trajectories leading toward hair shaft-, IRS-, and companion layer-associated states. **f,** Density distribution of young and aged IFC cells along the IFC pseudotime continuum (left) and global pseudotime distribution (right); ****: $p < 0.0001$, KS test.

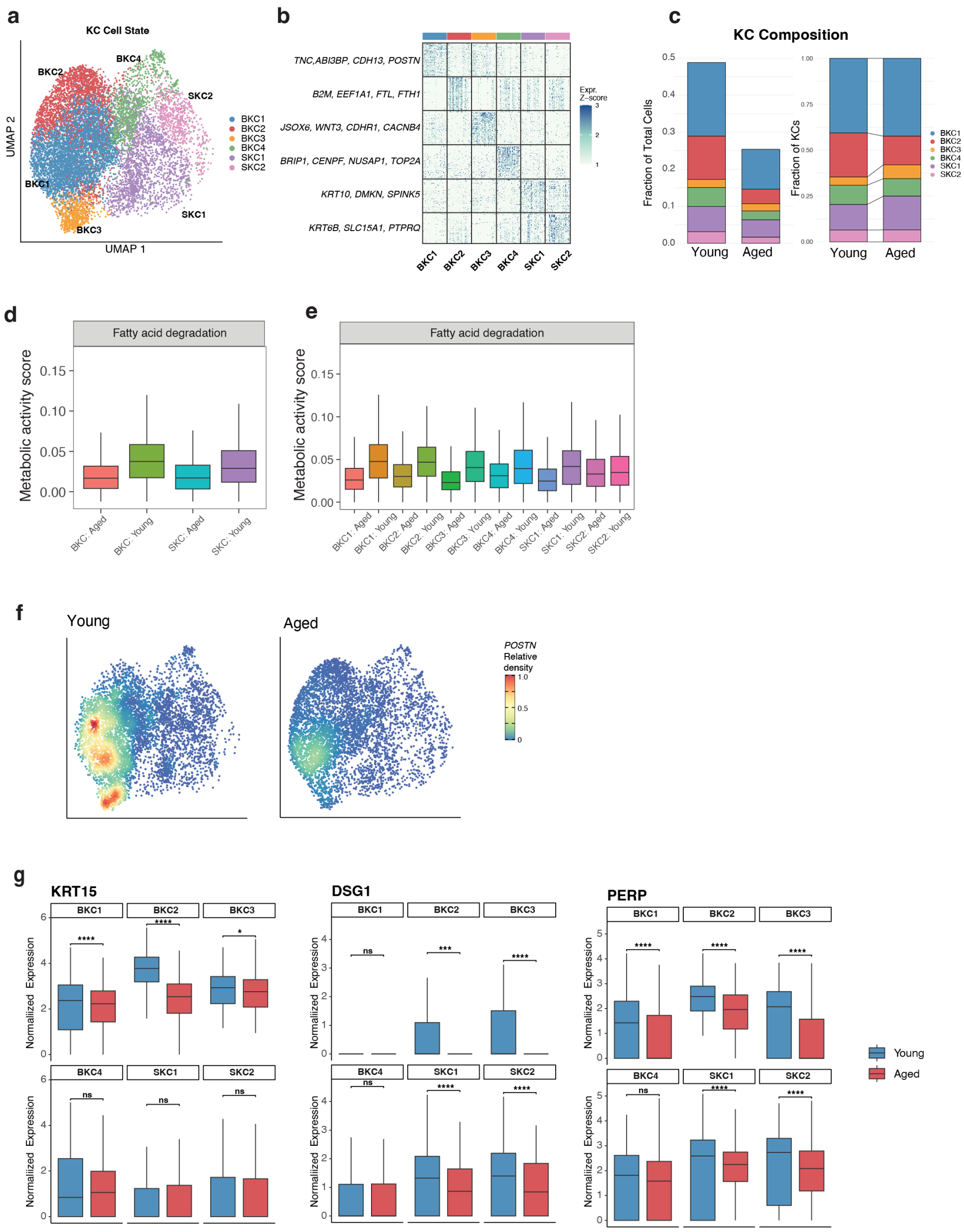

Figure S3. Keratinocyte subpopulation states and age-related functional shifts.

a, UMAP representation of keratinocytes (KCs) resolving two suprabasal subsets (SKC1-2) and four basal subsets (BKC1-4). **b,** Heatmap of KC state signature genes (scaled Z-scores) with representative marker genes listed on the left; each column corresponds to a state shown in **a**. **c,** Composition analysis comparing relative proportions of KC subpopulations between young and aged PSUs. Left: relative abundance normalized to total cells; right: relative abundance normalized to all KCs. **d,e,** Box plots showing mean expression of fatty acid degradation pathway genes in BKC and SKC populations (**d**) and their individual cell states (**e**)

stratified by age. **f,** Nebulosa density plots showing *POSTN* expression in young (left) and aged (right) KCs. **g,** Box plot showing expression z-scores of barrier protein genes across KC subsets stratified by age groups. Statistical significance was determined by two-tailed Wilcoxon rank-sum test; *: $p < 0.05$, ***: $p < 0.001$, ****: $p < 0.0001$; ns: not significant.

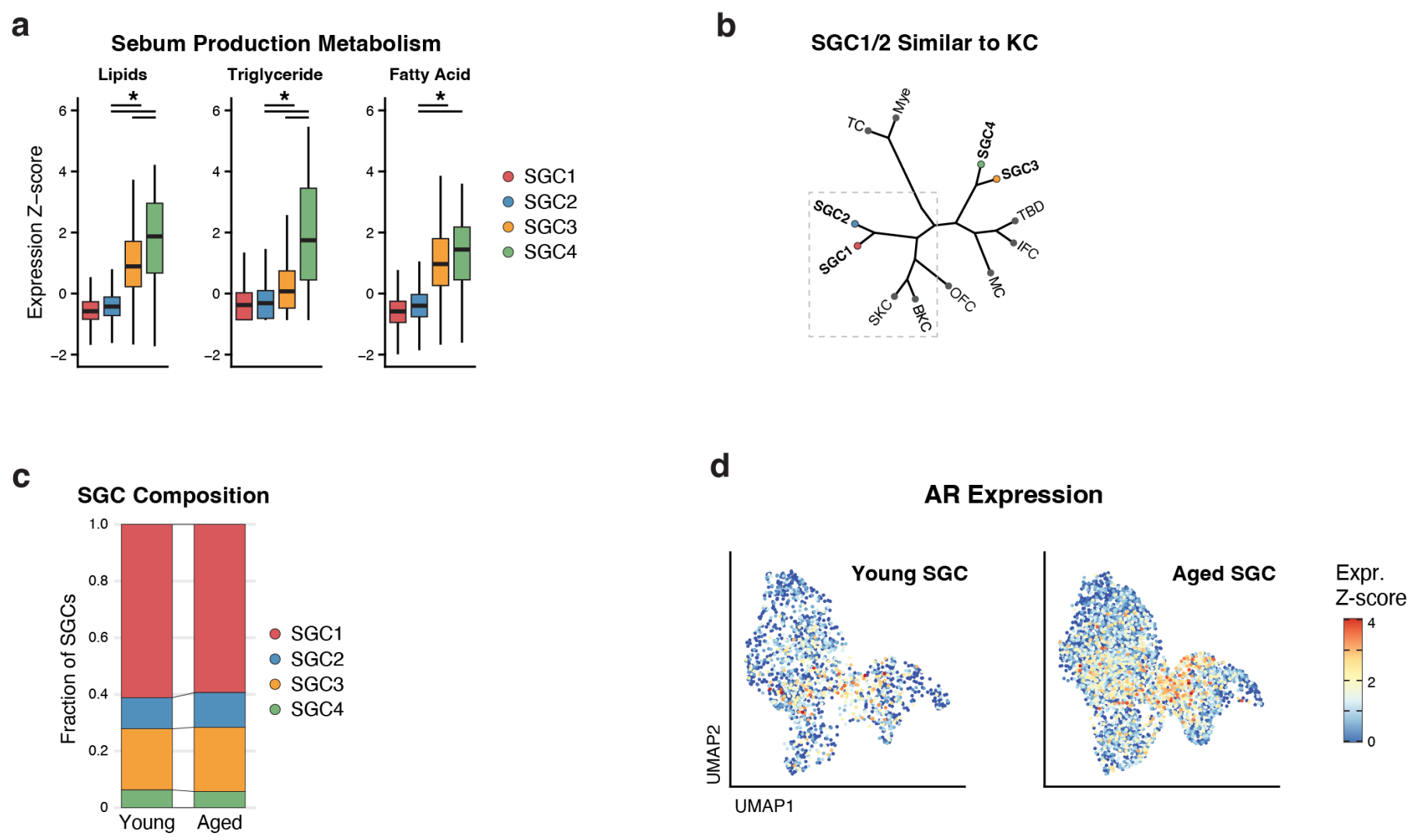

Figure S4. Additional characterization of sebocyte states and age-associated androgen signaling.

a, Box plot showing pathway activity scores for lipid, triglyceride, and fatty-acid metabolism across SGC states; ***: $p < 0.001$, by two-tailed Wilcoxon rank-sum test. **b,** Hierarchical tree based on Euclidean distance showing transcriptional similarity among SGC states and other PSU cell types. **c**, Composition analysis comparing relative proportions of SGC subpopulations between young and aged PSUs. **d,** Split-by-age UMAPs showing *AR* expression across sebocyte populations.

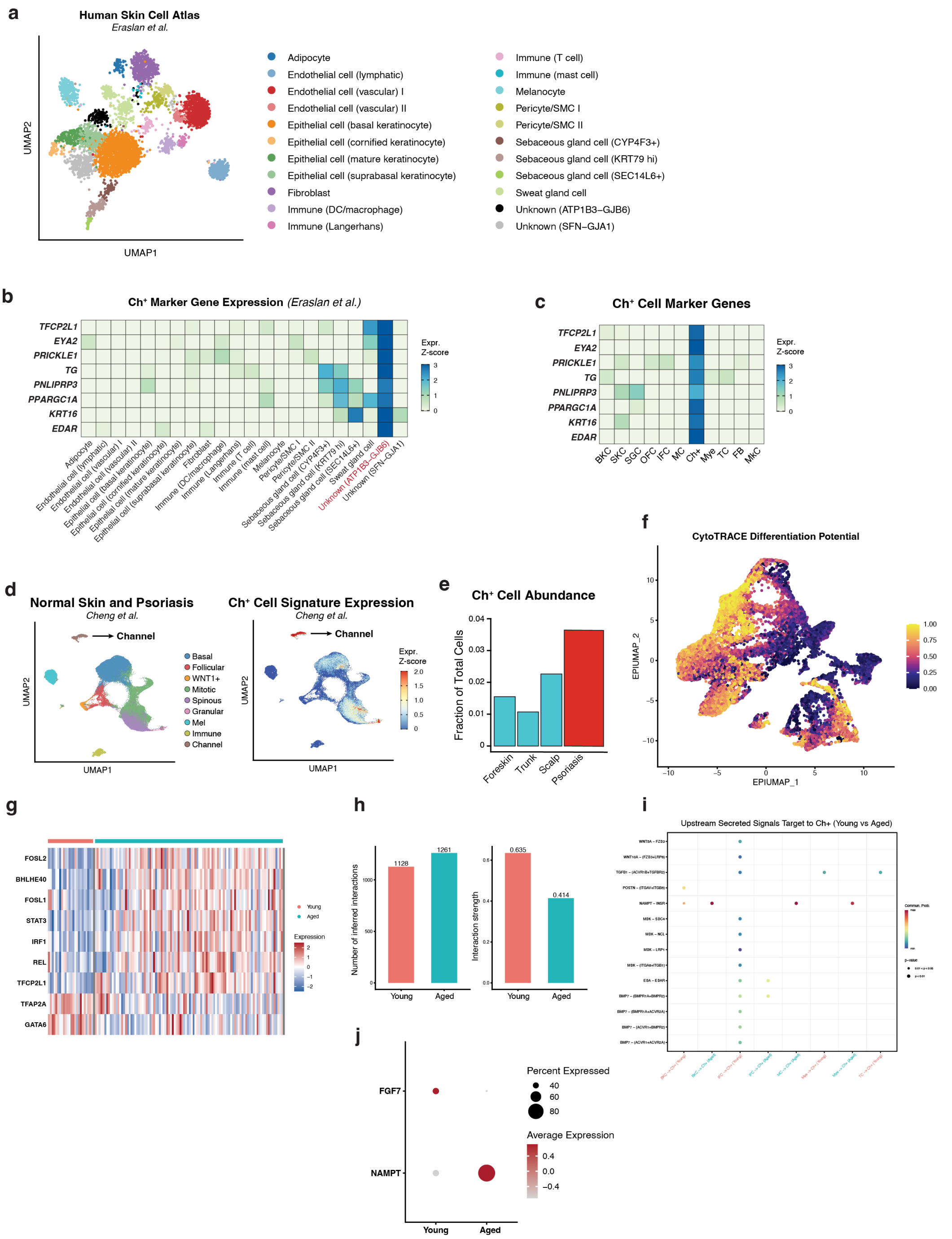

Figure S5. Validation, lineage inference, and signaling analyses of the channel⁺ epithelial state.

a, UMAP plot of reprocessed Human Skin Cell Atlas data showing cell types based on the original annotation by Eraslan *et al.* **b**, Heatmap showing the expression of representative Ch⁺ marker genes in the Human Skin Cell Atlas dataset. **c,** Heatmap of representative Ch⁺ marker genes in the current PSU dataset. **d**, Analysis of a published human psoriasis skin dataset (Cheng *et al.*): left, UMAP plot showing major skin cell types including a Channel cluster; right,

expression of Ch$^+$ signature genes. **e,** Bar plot showing the relative abundance of Ch$^+$ across tissue locations in Cheng *et al.* dataset. **f,** UMAP plot showing CytoTRACE scores across epithelial lineages. **g,** Heatmap showing SCENIC regulon activity scores of selected transcription factors in young and aged Ch$^+$ cells. **h,** Bar plots comparing the total number of inferred interactions (left) and overall interaction strength (right) originating from Ch$^+$ cells between young and aged conditions via CellChat analysis. **i,** Bubble plot of age-stratified CellChat analysis showing inferred incoming secreted signaling pathways received by Ch$^+$ cells. **j,** Dot plot showing the mean expression levels and percentage of cells expressing *FGF7* and *NAMPT* in Ch$^+$ cells across young and aged groups.

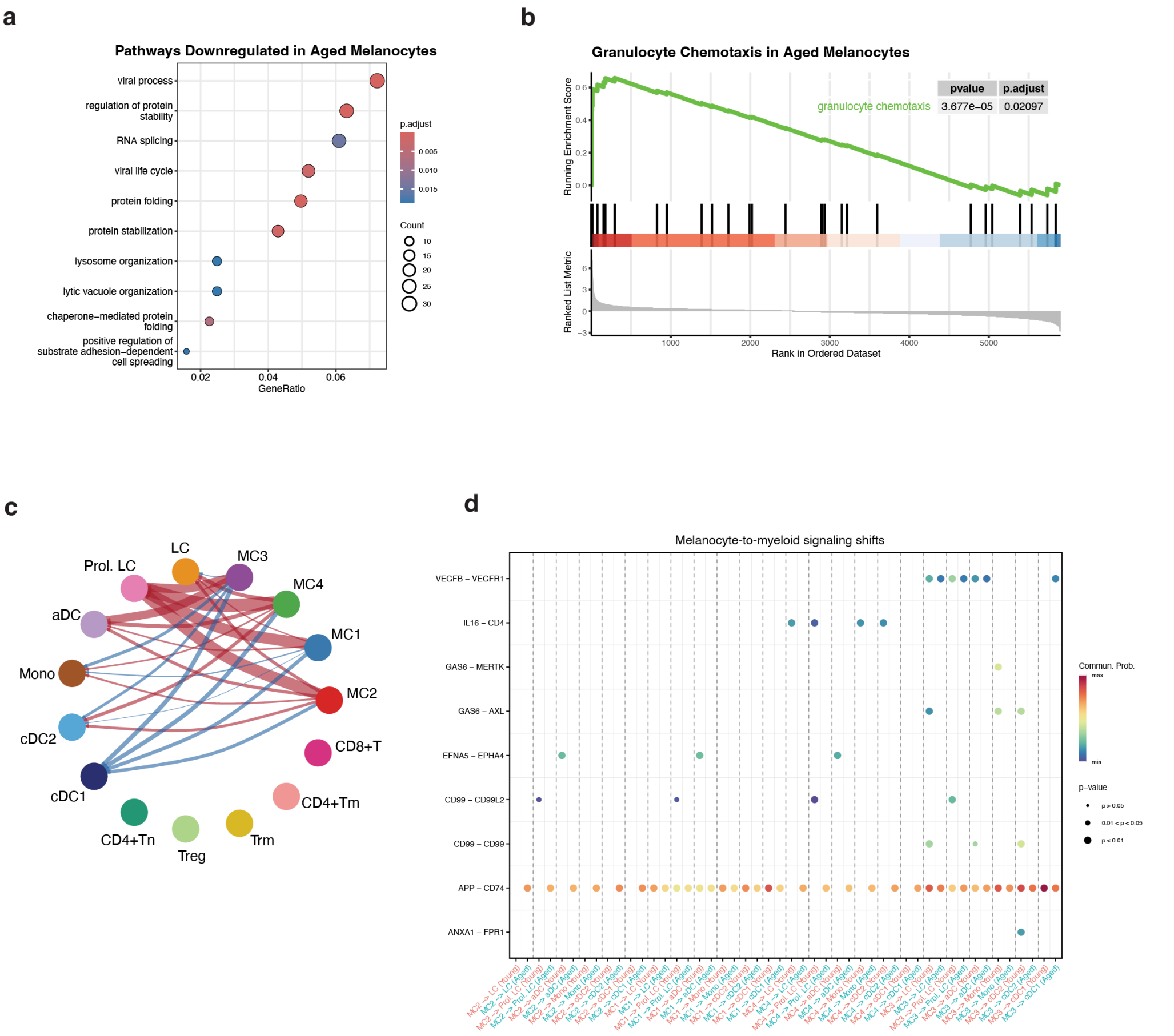

Figure S6. Functional remodeling and altered immune communication of aged melanocytes.

a, GO Biological Process enrichment analysis of genes downregulated in aged melanocytes. **b,** Gene Set Enrichment Analysis (GSEA) showing enrichment of granulocyte chemotaxis-associated programs in aged melanocytes. **c,** Differential CellChat interaction network comparing young and aged PSUs. Red lines indicate increased signaling strength, and blue lines indicate decreased signaling strength in aged PSUs. **d,** Bubble plot showing representative melanocyte-to-myeloid ligand-receptor interactions identified by CellChat analysis.

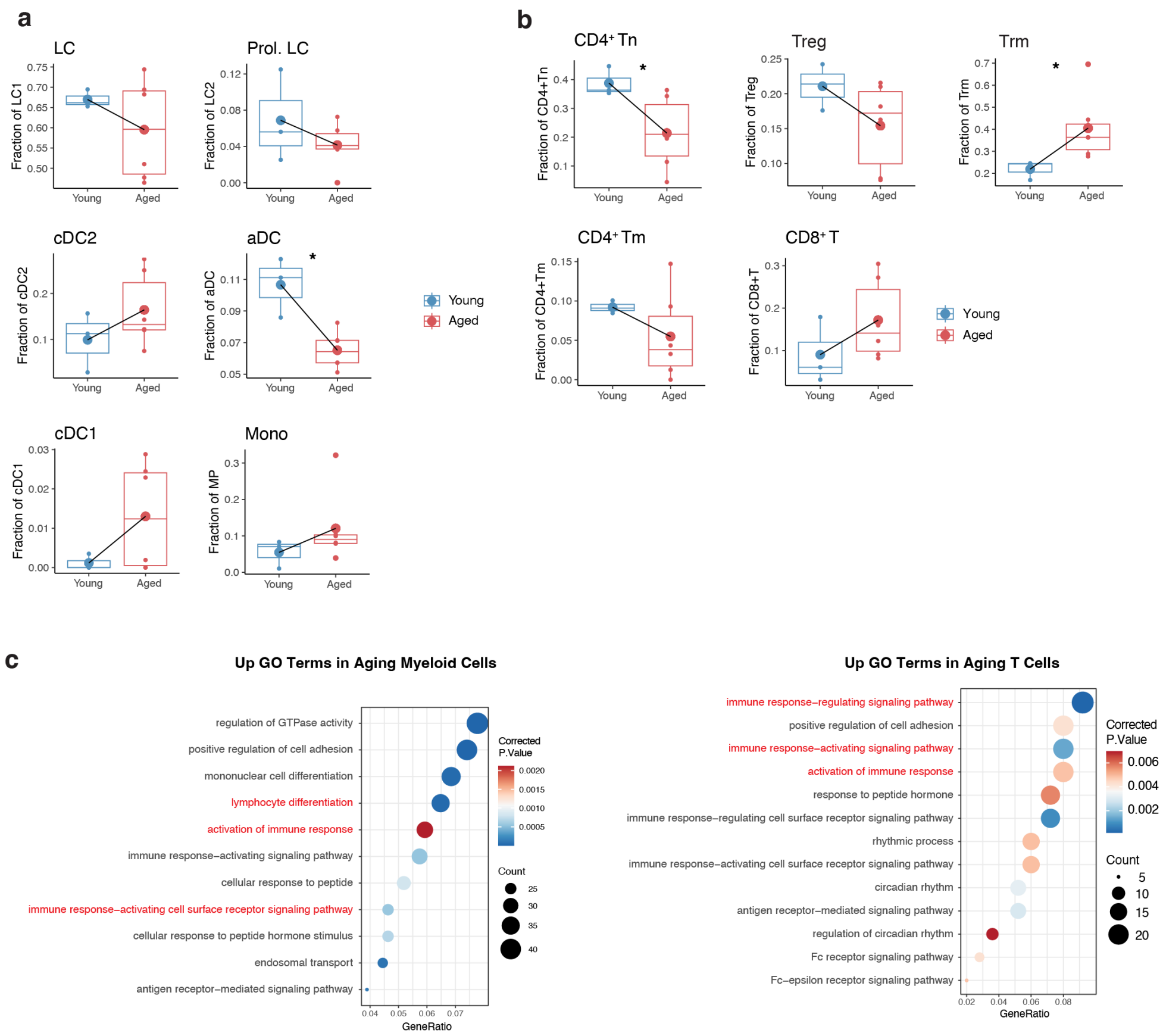

Figure S7. Immune cell subset composition and functional enrichment during aging.

a,**b,** Box plots showing per-donor (dot) quantification of cell abundance for myeloid (**a**) and T cell (**b**) states stratified by age (young, n = 3 donors; aged, n = 6 donors). *: $p < 0.05$, by two-tailed Wilcoxon rank-sum test; black lines connect group means. **c**, Dot plot showing top enriched GO Biological Process terms in aged myeloid cells (left) and T cells (right). Immune activation-related terms are highlighted in red. Benjamini-Hochberg adjusted p-values are shown.

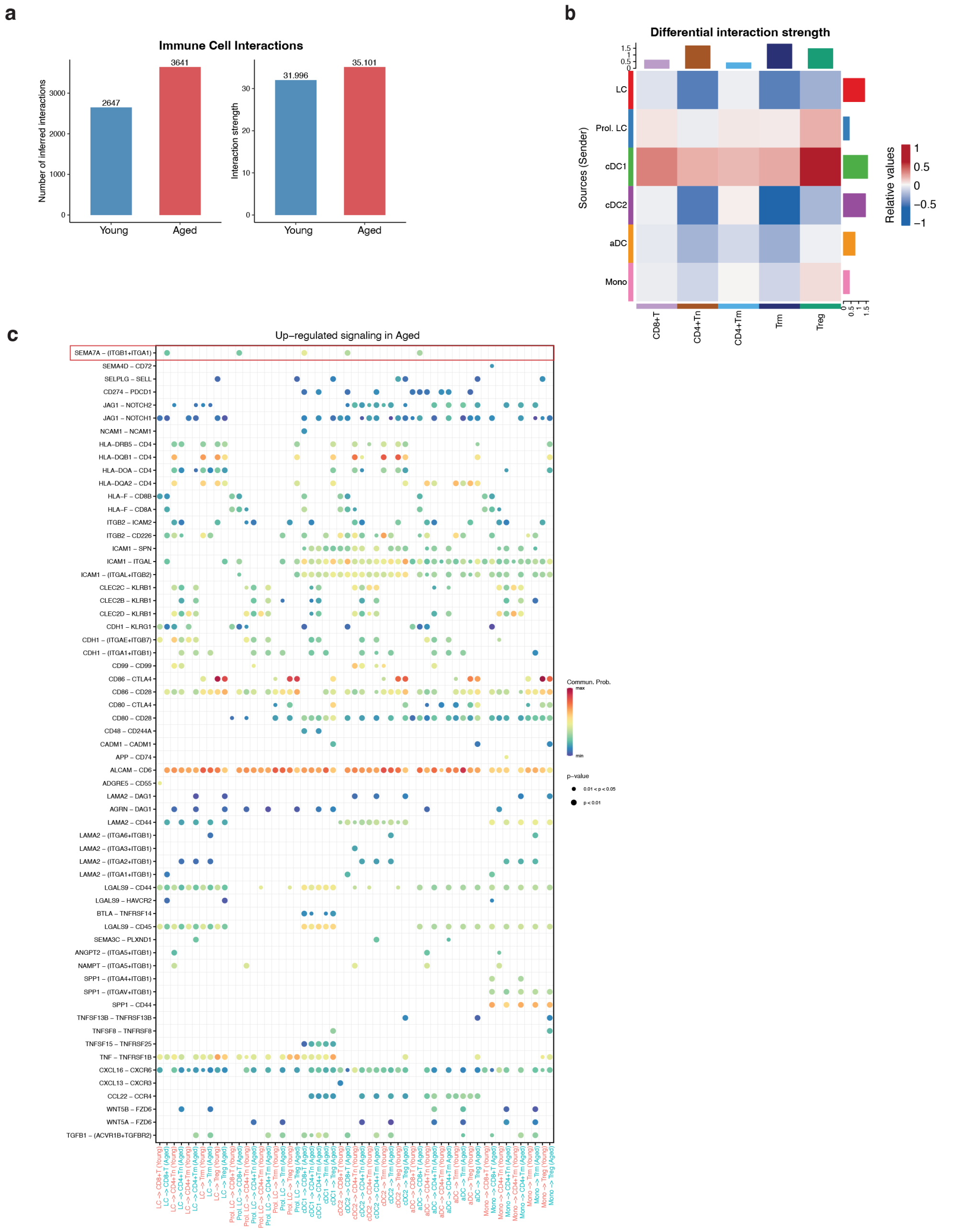

Figure S8. Global and immune-specific alterations in cell-cell communication with aging.

a, Bar plots showing CellChat-predicted ligand-receptor pair counts (left) and cumulative interaction strength (right) per age group. **b**, Heatmap showing CellChat-predicted differential interactions between aged and young samples. Color scale represents interaction strength. Rows represent ligand-sending cell populations; columns represent receptor cell populations. **c**, Dot plot showing aged sample-enriched interactions between myeloid immune cells and T cells. Dot color represents interaction probability.

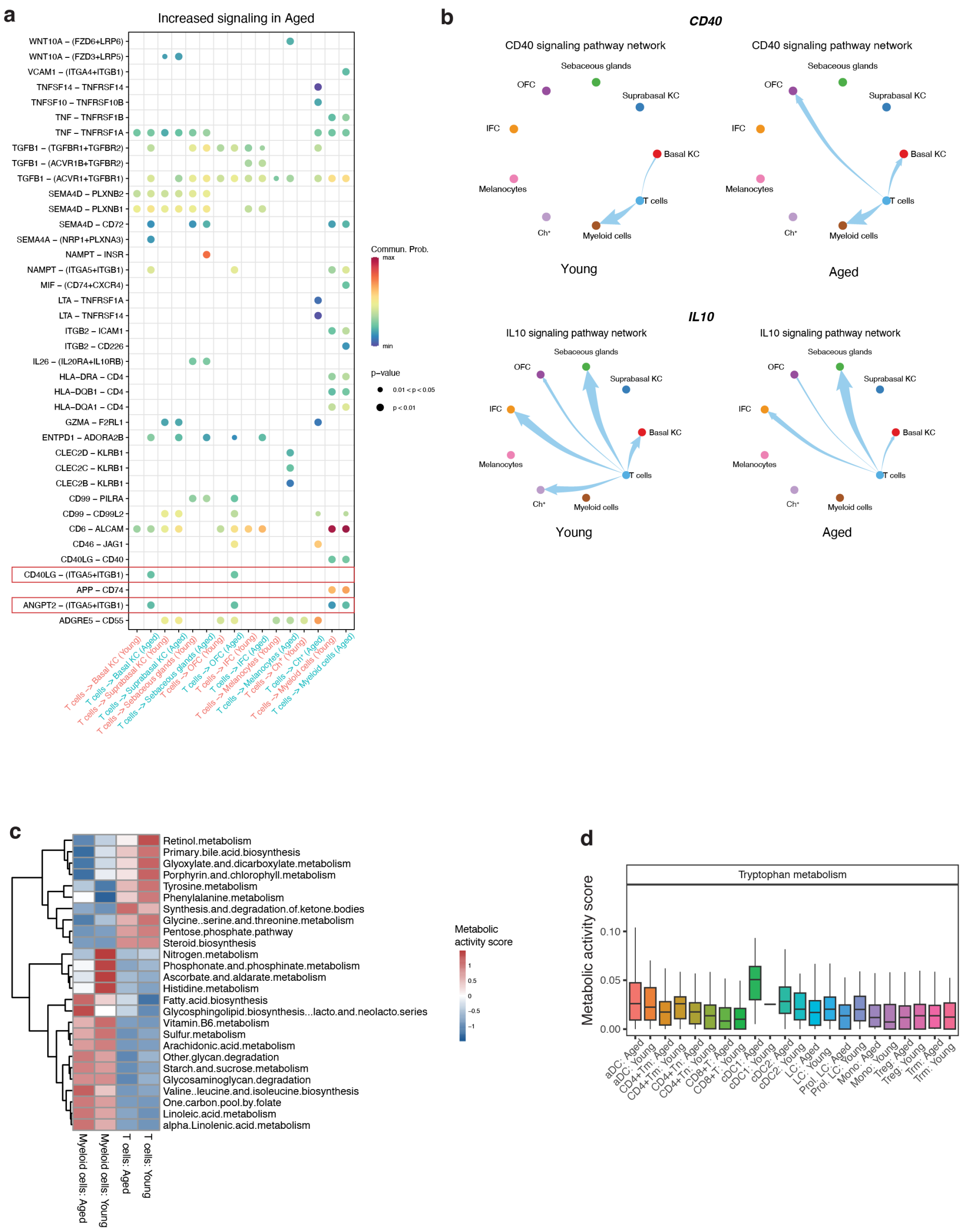

Figure S9. Age-remodeled intercellular signaling and immune cell metabolism.

a, Dot plot showing aged sample-enriched interactions between T cells and non-immune PSU cells. Dot color represents interaction probability. **b**, Predicted cell-cell communication networks showing ligand-receptor interactions from T cells to other PSU cell types through CD40 signaling (top) and IL-10 signaling (bottom) between young and aged samples. **c**, Heatmap showing metabolic pathways enriched in immune cells from aged and young samples. **d**, Box plot showing tryptophan metabolism pathway gene expression across immune cell subsets stratified by age groups.
